# Switchable supramolecular polycationic assemblies for tunable antibacterial strategies against antibiotic resistance

**DOI:** 10.1039/d5sc05035a

**Published:** 2025-10-07

**Authors:** Jia Chen, Xueqian Wang, Mengrui Zhang, Xue Wang, Ran Wang, Xinxing Lyu, Yunjian Xu, Xintian Shao, Luling Wu, Tony D. James

**Affiliations:** a School of Radiology, Shandong First Medical University & Shandong Academy of Medical Sciences Tai'an Shandong 271016 China chenjia@sdfmu.edu.cn; b Medical Science and Technology Innovation Center, Shandong First Medical University & Shandong Academy of Medical Sciences Jinan Shandong 250117 China shaoxintian@sdfmu.edu.cn; c State Key Laboratory of Analytical Chemistry for Life Science, School of Chemistry and Chemical Engineering, Nanjing University 163 Xianlin Avenue Nanjing 210023 China wllcyl@126.com; d Department of Chemistry, University of Bath Bath BA2 7AY UK

## Abstract

Bacterial resistance significantly hampers the efficacy of antibiotics in eradicating pathogens and treating infections. Here, we introduce an Adaptive Cationic Therapeutic Integrated (ACTI) system, a design strategy integrating pyridinium cationic membrane disruption and tunable antibacterial activity to address this challenge. ACTI leverages the assembled hyper-enriched cationic domains to enhance the destruction of bacterial membranes, while also enabling the on-demand deactivation of antibacterial activity through disassembly, thereby safeguarding biocompatibility. Additionally, ACTI facilitates the photodynamic inactivation of negatively charged photosensitizers (TPPS) by promoting the interaction between the photosensitizer and bacteria as well as aiding the transport of singlet oxygen. ACTI-loaded photosensitizers (TPPS@ACTI) exhibited potent antibacterial activity (>99% pathogen elimination) against methicillin-resistant *S. aureus* (MRSA) and *E. coli in vitro*, and the antibacterial efficacy was further validated using an MRSA-infected murine wound model. ACTI establishes a paradigm shift for the design of tunable antimicrobials that balance potency and biosafety in complex biological environments.

## Abstract 2

A POSS-based styrylpropylpyridinium iodide (PSPI) was synthesized. The addition of CB[8] or ADA enables switching of the antibacterial activity “ON” by assembling supramolecular polycations, or “OFF” by disassembly.

## Graphical abstract

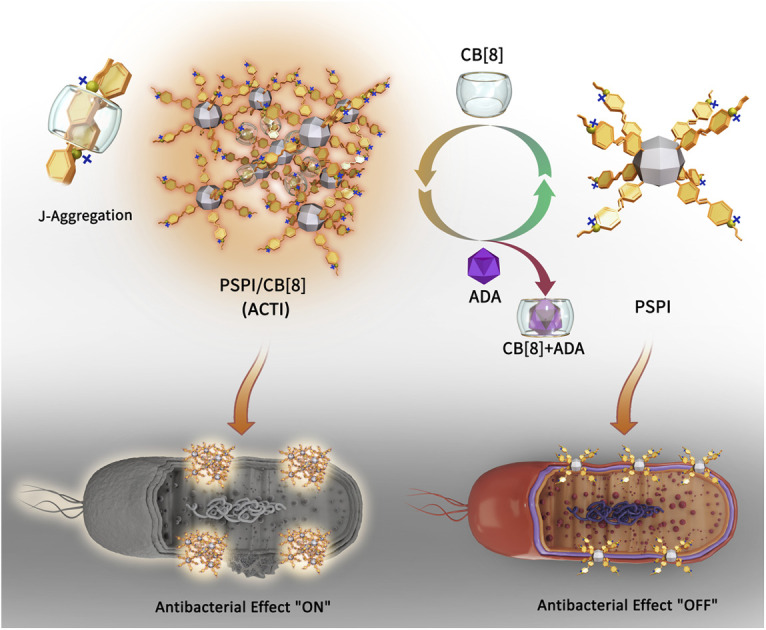

## 1 Introduction

Bacterial infections pose a significant global threat, resulting in millions of deaths worldwide and ranking as the second-leading cause of mortality.^1–3^ The primary strategy for combatting bacteria involves the use of antibiotics, which saves countless lives annually.^4,5^ Nevertheless, the widespread and excessive use of antibiotics has led to a pressing issue: the emergence of antibiotic resistance.^6^ In response, efforts have been directed to the development of new and more potent antibiotics.^7,8^ Nonetheless, the creation of new antibiotics involves complex and time-consuming chemical synthetic processes, while often resulting in unsatisfactory performance.^9,10^ Moreover, the widespread use of such novel antibiotics can inadvertently contribute to increased drug resistance.

To address this issue, researchers discovered that cationic compounds could spontaneously infiltrate and disrupt bacterial membranes, prompting the design of cationic oligomers and polymers (polycations) that exhibit reduced susceptibility to drug resistance.^11–13^ Specifically, positively charged pyridinium cations have been reported that can destroy bacterial membrane structures resulting in irreversible antibacterial activity, which is highly effective against bacterial resistance.^14–16^ Based on this, we rationally designed small molecular fluorescent pyridinium (4-bromostyrylpropylpyridium iodide, SPI) and then covalently conjugated it with POSS, forming POSS styrylpropylpyridium iodide (PSPI). Polyhedral oligomeric silsesquioxanes (POSSs) are inherent three-dimensional building blocks possessing eight antenna arms, making them valuable for constructing conjugated molecules with properties akin to those of oligomers.^17,18^ Nevertheless, oligomeric cations, such as PSPI, due to their relatively lower positive charge density and molecular weight compared to polycationics, result in a milder biocidal performance than the corresponding polycations.^19,20^

**Fig. 1:**
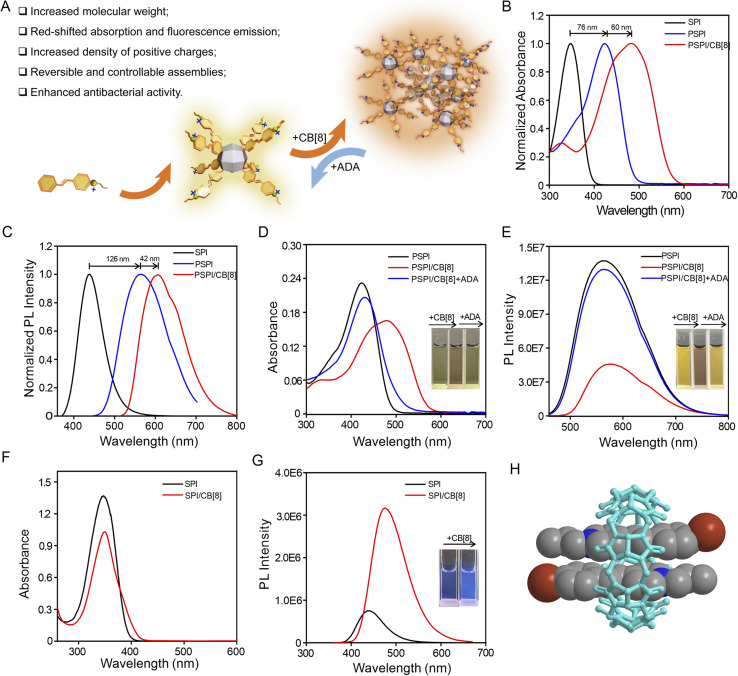
(A) Schematic diagram for the rational design of pyridinium cation based supramolecular antibiotics. The normalized UV-vis absorbance (B) and fluorescence spectra (C) of SPI, PSPI and PSPI/CB[8]. UV-vis absorbance (D) and fluorescence spectra (E) of 5 μM PSPI, 5 μM PSPI/CB[8] and PSPI/CB[8] + ADA; the inset photos show samples of PSPI, PSPI/CB[8] and PSPI/CB[8] + ADA under white light or a 365 nm UV lamp, respectively. The spectra of UV-vis absorption (F) and PL intensity (G) of SPI and SPI/CB[8] complex. The inset photo shows SPI and SPI/CB[8] under a 365 nm UV lamp. (H) Energy optimized model of SPI/CB[8] complexation in an aqueous environment simulated using Chem 3D and MM2 packages. The SPI compounds are presented as a space filling model, while CB[8] is a ball-and-stick model. Note: the iodide ions are omitted for clarity.

**Scheme 1:**
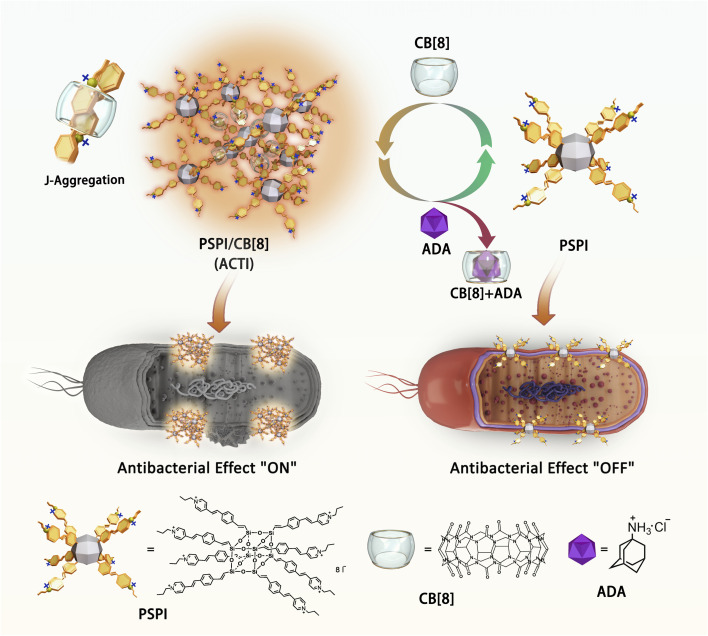
Schematic diagram for switchable antibacterial activity of pyridinium-based supramolecular polycations.

To enhance the antimicrobial activity of oligo-cationic compounds and address the aforementioned challenge—eradicating drug-resistant bacteria without promoting further resistance, we developed an Adaptive Cationic Therapeutic Integrated (ACTI) system. This was achieved by forging the oligo-cationic building blocks into a higher-order polycationic architecture, utilizing cucurbit[8]uril (CB[8]) as a molecular junction through a supramolecular assembly strategy. Cucurbit[8]uril (CB[8]) is a macrocyclic host that is widely exploited to bind guest molecules and construct supramolecular assemblies for biomedical applications.^21–25^ Through the encapsulation of CB[8], oligomeric cations with poor antibacterial performance could be transformed into high molecular-weight polycations with enhanced charge density, thereby enhancing their antibacterial efficacy (Fig. 1A). For example, an environmentally friendly antibiotic using cucurbit[8]uril-mediated branched polyethylenimine has been developed, endowing it with *in situ* activatable antibacterial activity.^19^ Significantly, the pyridinium cation structure we have developed binds strongly with CB[8] due to its hydrophobic and planar chemical structure and its positive charge characteristics.^26,27^ We hypothesize that by using supramolecular assembly, the positive charge density and molecular weight of the assemblies will increase, leading to enhanced antibacterial activity due to increased cell membrane affinity. Additionally, its exceptional and adjustable optical properties and straightforward synthesis provide advantages in facilitating antimicrobial activity and elucidating the antimicrobial mechanism. More importantly, compared to traditional antimicrobial reagents, such as antibiotics that are “always-ON”, such supramolecular cationic integration consisting of PSPI/CB[8] assemblies undergoes disentanglement upon the addition of a competitive guest molecule, amantadine hydrochloride (ADA), and reverts to its original form—the oligomeric PSPI (Scheme 1). This disassembly is accompanied by a reduction in molecular weight and a decrease in local positive charge density, leading to reduced antibacterial performance, thus generating a reversible antibiotic with tunable antibacterial properties thereby avoiding the occurrence of drug resistance.

## 2 Results and discussion

### 2.1 Design and synthesis of PSPI and its assembly with CB[8]

**Scheme 2:**
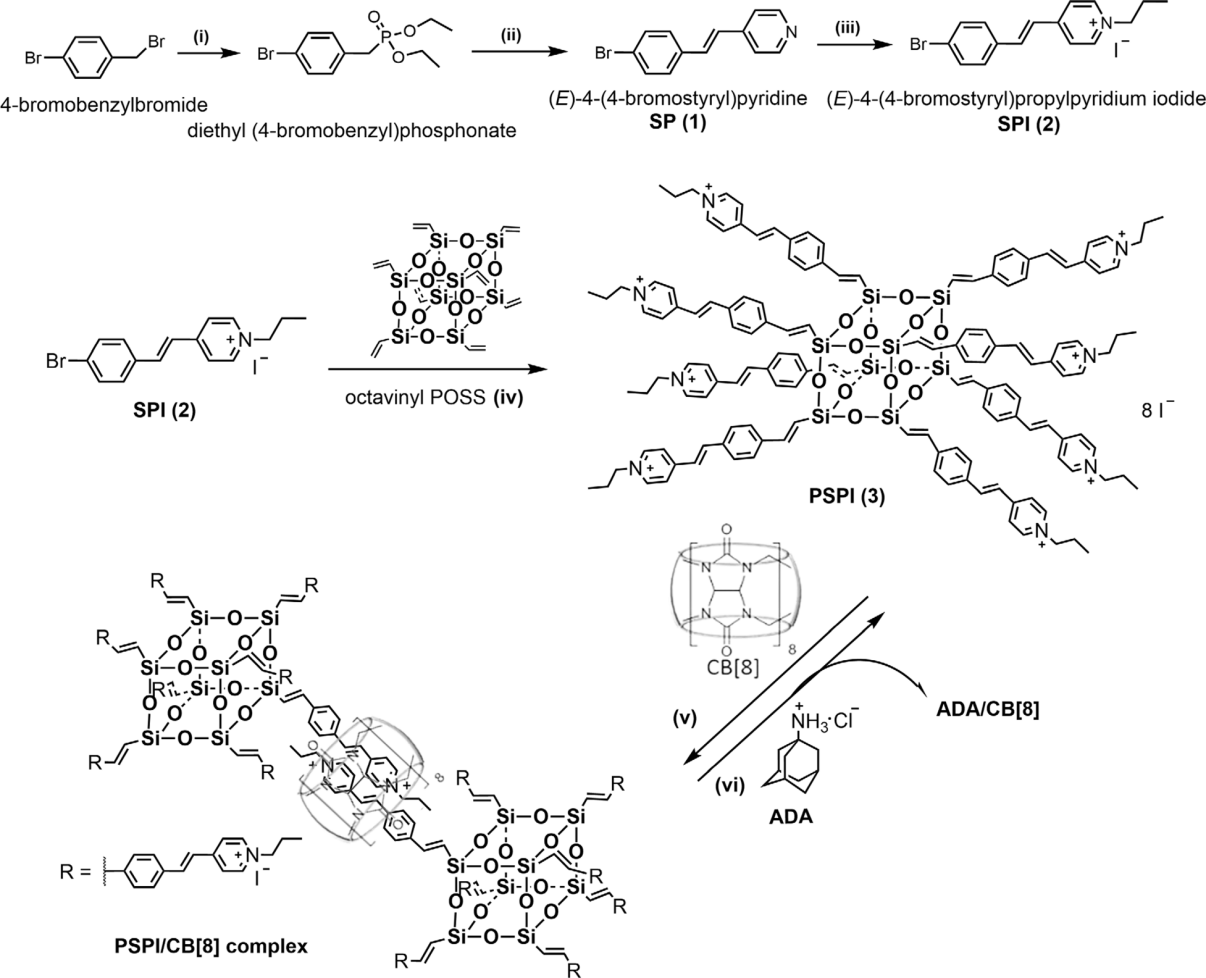
The synthesis and construction of PSPI and PSPI/CB[8] assembly.

First, 4-styrylpyridinium iodide (SPI) was designed and synthesized (Scheme 2). This was followed by conjugation with an octavinyl POSS building block *via* Heck coupling, generating an octatopic styrylpyridinium guest molecule (PSPI). The chemical structures of SPI and PSPI were confirmed using ^1^H, ^13^C NMR and mass spectroscopy (Fig. S1–S9). The proton at 7.71 ppm in the ^1^H NMR spectrum of SPI (Fig. S5) was assigned to the proton at the 1-position of the benzene near the bromide group, shifting upfield to 7.45 ppm in the ^1^H NMR spectrum of PSPI (Fig. S7), while the total number of protons of PSPI in the aryl region was consistent with the calculated number of protons. In addition, the ^13^C NMR spectra and mass characterization also validate the successful conjugation of SPI with octavinyl POSS (Fig. S6 and S9).

### 2.2 Characterization and determination of PSPI and PSPI/CB[8]

Next, we investigated the photophysical and photochemical properties of SPI, PSPI and the supramolecular assembly of PSPI with CB[8]. As shown in Fig. S10, the fluorescence of SPI is weak in aqueous solution due to the relatively short length of π conjugation and the electron-withdrawing effect of the bromine atom. In contrast, PSPI exhibited a remarkable fluorescence enhancement, and its absorbance and emission underwent significant bathochromic shifts of approximately 76 nm (Fig. 1B) and 126 nm (Fig. 1C), respectively. This is attributed to the length of π conjugation which was significantly extended through the cubic skeleton^28,29^ and the reduced electron-withdrawing effect due to the conjugation between the SPI arms and octavinyl POSS. Additionally, PSPI could readily dissolve in common polar solvents, such as DMSO, DMF, EtOH, MeOH, water and PBS, suggestive of good processability and ease of post functionalization (Fig. S11). As designed, the PSPI and CB[8] exhibit strong supramolecular interactions, which is evident in the UV-vis absorbance (Fig. 1B) and fluorescence spectra (Fig. 1C). Both the absorption and fluorescence peaks of the PSPI-based supramolecular assembly broadened and experienced a bathochromic shift upon the addition of CB[8]. This phenomenon is caused by J-aggregation, of which J-dimers of the (*E*)-styrylpyridinium arms of PSPI within the CB[8] cavity are formed due to multiple noncovalent interactions including the hydrophobic effect, ion–dipole interactions, and host-stabilized charge-transfer interactions.^26,27,30^ The observation of SPI J-aggregation within the cavity of CB[8] also corroborates this conclusion (Fig. 1F and G). However, negligible changes were observed for PSPI upon adding CB[7] (Fig. S12), suggesting that PSPI J-dimer aggregations only occur within the CB[8] macrocycles. On the other hand, the PSPI/CB[8] supramolecular assemblies dissociate when an excess of ADA (5 equiv.) is added, leading to the subsequent restoration of the absorption and fluorescent emission of free PSPI, as indicated in Fig. 1D and E. These findings confirm the potential of ADA as an efficient disassembly agent for PSPI/CB[8] supramolecular assemblies.

**Fig. 2:**
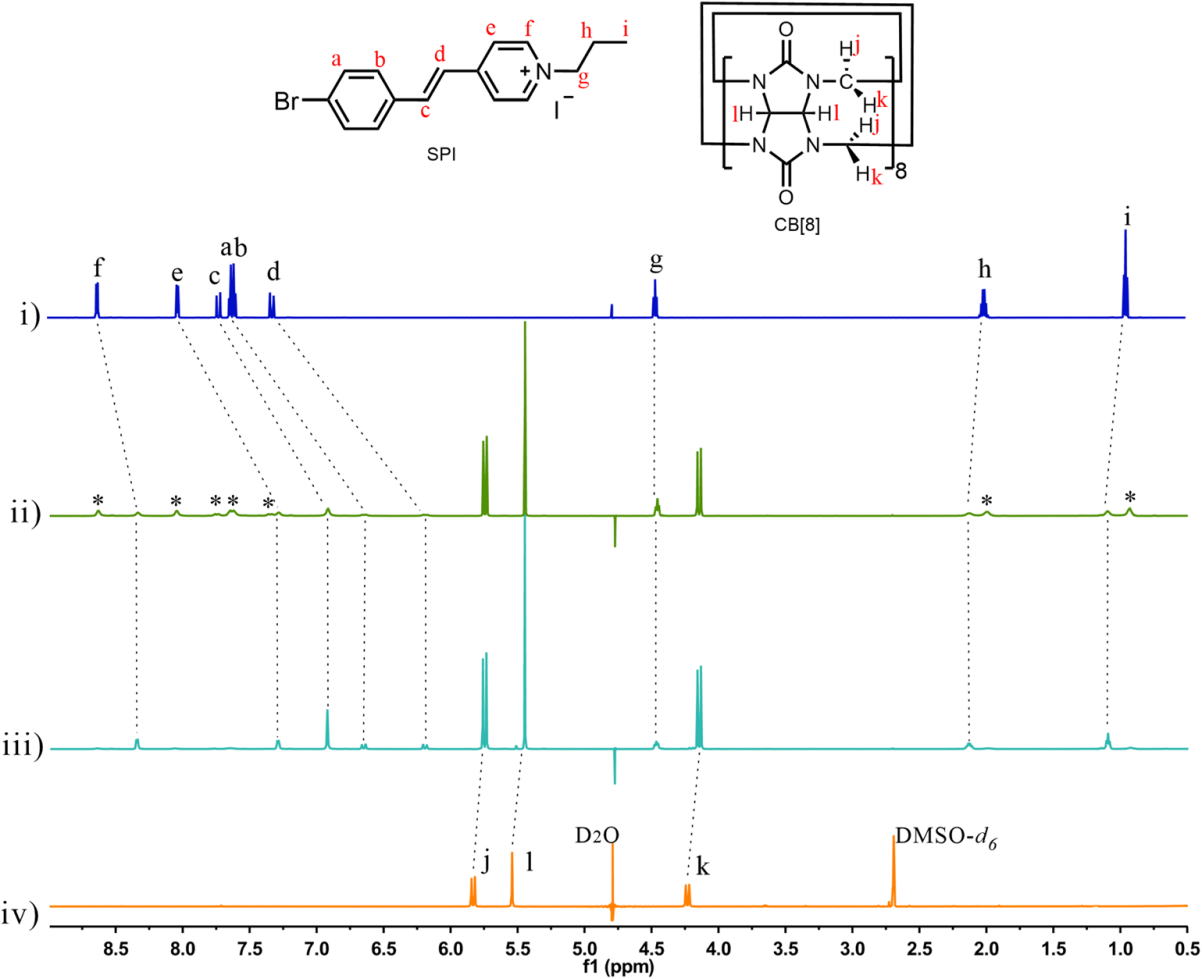
^1^H NMR spectra of (i) free SPI, (ii) SPI with the addition of 0.25 equiv. of CB[8] (SPI : CB[8] = 4 : 1, resonances of free SPI are marked with *), (iii) SPI with the addition of 0.5 equiv. of CB[8] (SPI : CB[8] = 2 : 1) and (iv) free CB[8] in D_2_O. (600 MHz, 298 K, D_2_O). About 10% DMSO-*d*_6_ was added into free CB[8] (iv) to meet the NMR test requirements and obtain a high quality spectrum. Note: efficient water suppression approaches were implemented for these NMR titration experiments.

Subsequently, we investigated the stoichiometry and binding between CB[8] (host) and SPI (guest). ^1^H NMR titrations were used to confirm the host–guest complexation between SPI and CB[8]. As shown in Fig. 2, upon addition of 0.25 equiv. CB[8] to SPI solution (SPI : CB[8] = 4 : 1), the protons in the aromatic part of SPI are divided into two sets of signals, where one signal undergoes an up-field shift and the other remains unaffected. When the concentration of CB[8] is 0.5 times that of SPI (SPI : CB[8] = 2 : 1), the peaks corresponding to the SPI protons were broadened. In particular, the signals for protons in the aromatic region of the SPI experience obvious up-field shifts from 8.64 to 8.35 (Δ*δ* = 0.29 ppm), from 8.04 to 7.30 (Δ*δ* = 0.74 ppm), from 7.74 to 6.93 (Δ*δ* = 0.81 ppm), from 7.64 to 6.67 (Δ*δ* = 0.97 ppm), and from 7.34 to 6.22 (Δ*δ* = 1.12 ppm), respectively. Besides, the detailed binding stoichiometry and binding affinity of SPI with CB[8] were further investigated using isothermal titration calorimetry (ITC). As illustrated in Fig. S13A and S13B, the ITC results reveal a 2 : 1 stoichiometry for the complexation between SPI and CB[8] with a binding constant of 5.4 × 10^5^ M^−2^, which is consistent with the previously reported binding stoichiometry and affinity between styrylpyridinium and CB[8].^26,27^ Additionally, the results of theoretical simulations also suggest that two SPI guest molecules were included within a CB[8] cavity with antiparallel orientation, forming 2 : 1 SPI/CB[8] J-dimer complexes (Fig. 1H). Similarly, the ^1^H NMR titration also confirmed the host–guest complexation between PSPI and CB[8] (Fig. S14). Upon addition of CB[8] to PSPI, the protons in the aromatic part of PSPI shift to lower ppm due to the shielding effect by CB[8], indicating the successful host–guest inclusion of PSPI with CB[8] and the CB[8]-induced J-aggregation process. As indicated in Fig. 2 and S13, when all SPI arms of PSPI were encapsulated by CB[8], the optimal binding ratio of PSPI/CB[8] was 1 : 4, because each PSPI molecule contains eight SPI arms and two SPI arms coordinate with one CB[8] macrocycle.

**Fig. 3:**
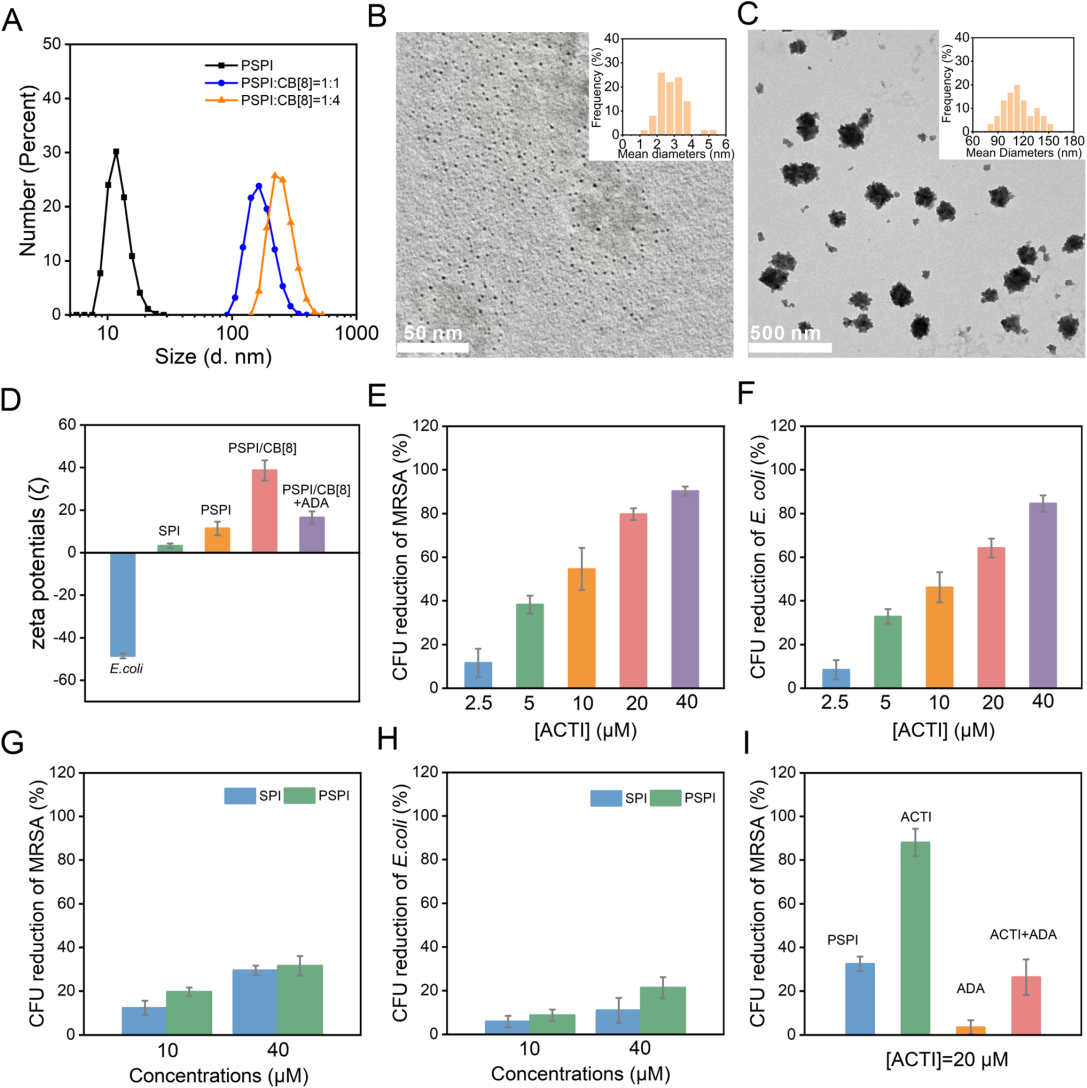
(A) DLS of PSPI upon adding different ratios of CB[8]. Typical TEM images of PSPI (B) and the PSPI/CB[8] assemblies (C). (D) Zeta potentials (*ζ*) of *E. coli*, SPI, PSPI, PSPI/CB[8] and PSPI/CB[8] + ADA, respectively. CFU reduction of MRSA (E) and *E. coli* (F) treated with different concentrations of ACTI. CFU reduction of MRSA (G) and *E. coli* (H) treated with SPI and PSPI at concentrations of 10 and 40 μM, respectively. (I) CFU reduction of MRSA treated with PSPI, ACTI, ADA and ACTI with ADA, respectively. The concentration of ACTI was assumed to be the same as that of PSPI. All experiments were repeated independently three times, and values are means ± SD (*n* = 3). * and ** indicate *P* < 0.05 and 0.01 compared to other groups using Student's *t*-test, respectively.

We then investigated their sizes, morphologies, and dispersion stabilities. The sizes were characterized in aqueous solutions and a dried state using dynamic light scattering (DLS) and transmission electron microscopy (TEM), respectively. As illustrated in Fig. 3A, the sizes indicated by the hydration radius of PSPI nanodots alone was about 10.8 nm, and the hydration radius of the complexes are enhanced with an increasing amount of CB[8], suggesting that PSPI gradually forms larger supramolecular assemblies due to host–guest interactions with CB[8]. In addition, solid state TEM could also be used to support the DLS measurements. The pristine PSPI nanodots were about 2.9 nm, while sizes of the formed supramolecular assemblies were 114.4 nm on average (Fig. 3B and C). The particle sizes characterized by dynamic light scattering (DLS) were slightly larger than those determined by TEM. This discrepancy can be attributed to the fact that DLS measures the hydrodynamic radius, which includes the hydration layer surrounding the supramolecular assemblies, whereas TEM provides measurements of these structures in a dehydrated state. In addition, the obtained supramolecular assemblies exhibited good water stability and they were found to remain stable for at least 7 days as determined by DLS measurements (Fig. S15). Both the size and density of positive charges ensure that these supramolecular assemblies possess a remarkable antimicrobial activity. Previous literature has shown that higher positive charge densities result in higher antibacterial efficiency, and different charge densities are reflected as variations in zeta potentials (*ζ*).^19^ Therefore, we measured the zeta potentials (*ζ*) to establish the charge densities of SPI, PSPI, PSPI/CB[8] and *E. coli* (Fig. 3D). The results revealed that after PSPI assembled with CB[8] into polycations, the zeta potentials of the assemblies increased, indicating that the densities of positive charges increased, suggesting an enhanced cell membrane disruptive capability and promising antibacterial activity. Based on the obtained experimental data, we therefore propose that the PSPI/CB[8] assemblies are supramolecular assembled polycations. Owing to the tunability of their cationic charge density, they can be categorized as an adaptive cationic therapeutic integrated (ACTI) system for the subsequent *in vitro* and *in vivo* investigations. We constructed ACTI from PSPI and CB[8] in an optimal molar ratio of 1 : 4, meaning that each ACTI assembly unit contains one PSPI molecule and four CB[8] molecules on average. Therefore, the ACTI concentration is four times the CB[8] concentration and can be considered equivalent to the PSPI concentration. In this study, the concentration of ACTI was assumed to be the same as that of PSPI.

### 2.3 *In vitro* antibacterial activity

We then evaluated the antibacterial activity of the pyridinium-based polycations with methicillin-resistant *Staphylococcus aureus* (MRSA) and *Escherichia coli* (*E. coli*), respectively. Colony forming unit (CFU) reduction was used to measure the antibacterial performance.^31,32^ Initially, we investigated the dose-dependent antibacterial activity of ACTI. As shown in Fig. 3E and F, a reduction of CFU correlated with an increased concentration of ACTI (2.5, 5, 10, 20 and 40 μM), and about 90.3% of MRSA and 84.6% of *E. coli* were inactivated by 40 μM ACTI, respectively. The trend of enhanced antibacterial effectiveness with higher concentrations aligns with previous literature,^32^ suggesting that the antibacterial performance improved with the increased concentration of polycations. ACTI exhibited an improved antibacterial effect against MRSA over *E. coli*, which is due to the inherent cell membrane differences, where Gram-negative bacteria (*E. coli*) possess a thicker and more complex cell membrane than Gram-positive bacteria (MRSA).^33^ In contrast, the antibacterial performance of SPI and PSPI were significantly lower than that of ACTI, suggesting the importance of a high density and concentration of cations for bacterial inactivation (Fig. 3G and H). As the supramolecular polycations could be disassembled into PSPI by a more competitive guest ADA, we then determined the antibacterial reversibility. As shown in Fig. 3I, after adding excess ADA, the antibacterial efficiency of ACTI was reduced from 88% to 26.4%, suggesting that the antibacterial ability of ACTI could be switched “OFF” by ADA.

**Fig. 4:**
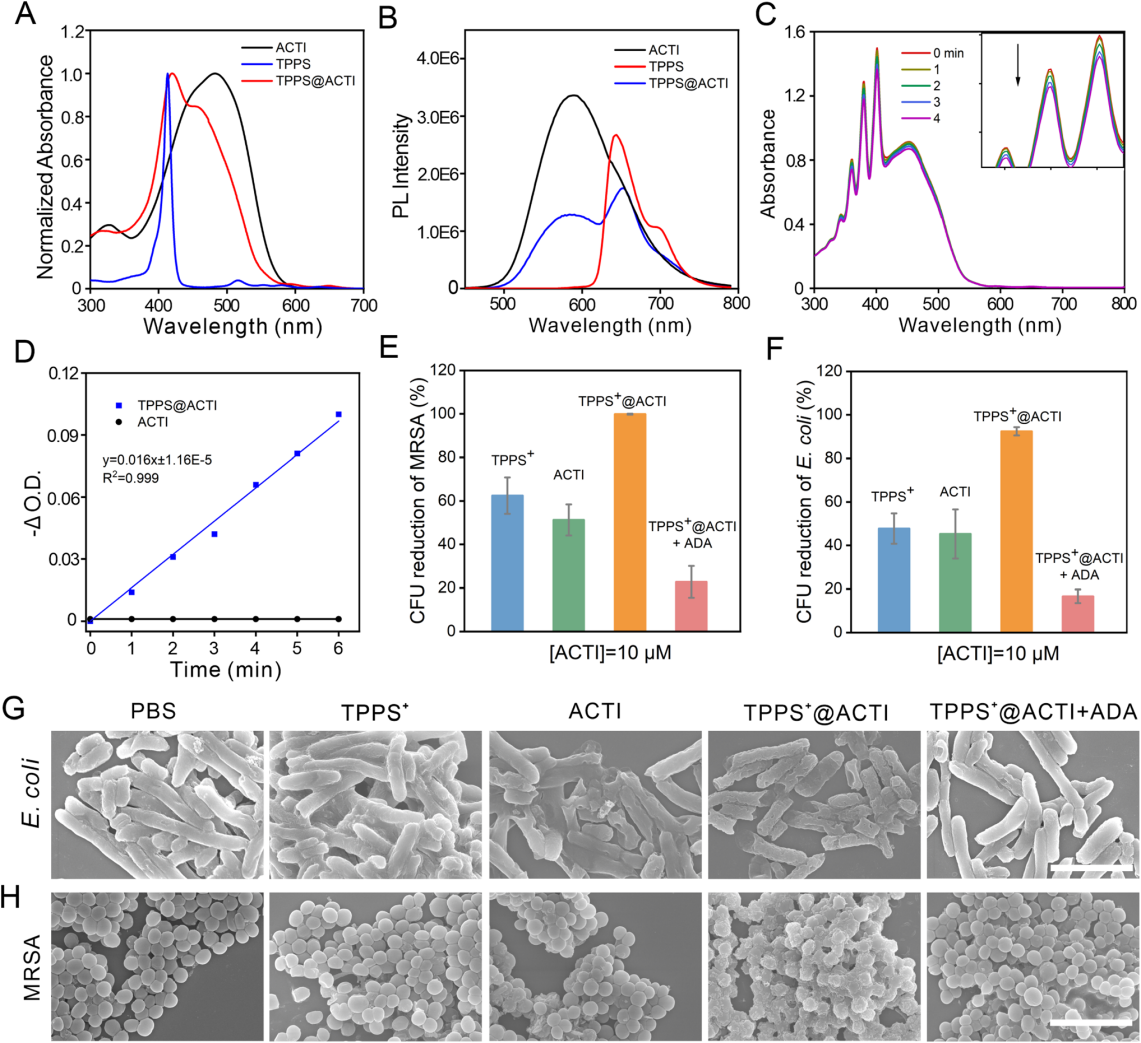
The normalized UV-vis absorbance (A) and fluorescence spectra (B) of ACTI (Ex = 460 nm), TPPS (Ex = 520 nm) and TPPS@ACTI (Ex = 460 nm). (C) The UV-vis absorbance spectra of TPPS@ACTI with the indicator ABDA under white light irradiation (400–700 nm, 50 mW cm^−2^). (D) The O.D. changes (−ΔO.D.) of ACTI and TPPS@ACTI against the time of white light illumination. The CFU reduction of MRSA (E) and *E. coli* (F) treated with TPPS^+^, 10 μM ACTI, 10 μM TPPS^+^@ACTI and 10 μM TPPS^+^@ACTI + ADA, respectively. Typical scanning electron microscopy (SEM) images of *E. coli* (G) and MRSA (H) with different treatments. All experiments were repeated independently three times; values are means ± SD (*n* = 3). Note: 10 μM TPPS@ACTI contains 6.4 μM TPPS and also 6.4 μM TPPS in the control groups. All groups containing TPPS were illuminated (400–700 nm, 50 mW cm^−2^) and marked as TPPS^+^. Scale bars: 4 μm.

*Meso*-Tetra(4-sulfonatophenyl)porphyrin (TPPS) is a porphyrinoid photosensitizer with good water solubility and remarkable singlet oxygen generation ability (*Φ* = 0.6 in water),^34,35^ while its antibacterial performance is very weak, due to the anionic nature of TPPS, which prevents it from effectively binding to negatively charged bacterial cell membranes. Previously, we confirmed that ACTI exhibits strong interactions with cell membranes. Inspired by this, we conceived combining the advantages of ACTI and TPPS to achieve a complementary and synergistic effect: ACTI adsorbs onto the bacteria and disrupts the cell membrane, while TPPS efficiently generates singlet oxygen for photodynamic inactivation (PDI), thereby achieving synergistic antibacterial activity. It has been reported that supramolecular assemblies with positively charged pyridinium salts could be applied to load negatively charged molecules, such as macromolecular DNA and small-molecule carboxylate drugs, using interactions between the positive/negative charges and hydrophobic interactions.^36,37^ These previous examples provided a theoretical basis for the loading of ACTI with TPPS. Thus, we performed the loading of the supramolecular polycations (ACTI) with TPPS; successful loading was characterized and validated by UV-vis absorbance (Fig. 4A) and fluorescence emission measurements (Fig. 4B). The loading efficiency of ACTI for TPPS was calculated to be 64% (SI, Fig. S16 and S17). Subsequently, the singlet oxygen (^1^O_2_) generation was measured with 9,10-anthracenediyl-bis(methylene)dimalonic acid (ABDA). As shown in Fig. 4C and D, after loading the TPPS photosensitizer, the ^1^O_2_ generation of TPPS@ACTI exhibited a significant enhancement, resulting in boosted antibacterial efficiency. Subsequently, the antibacterial efficiency of TPPS and the TPPS@ACTI complex were investigated. As illustrated in Fig. 4E and F, the antibacterial activity of TPPS@ACTI was significantly enhanced when compared with TPPS alone, benefiting from the membrane-disrupting effect of ACTI. 10 μM TPPS@ACTI exhibited a 37% improvement in anti-MRSA efficacy compared to TPPS alone. As a contrast, TPPS alone does not exhibit significant photodynamic anti-bacterial activity, which is attributed not only to its poor binding affinity to the bacteria cell membranes,^38^ but also to the π–π stacking induced aggregation-caused quenching (ACQ) of the photosensitizer.^39^ Additionally, as expected, ADA can also effectively disassemble the TPPS@ACTI complex thus lowering its antibacterial performance. Moreover, the CFU observed from photographs of LB agar plates were consistent with the measured antibacterial efficacy (Fig. S18).

### 2.4 Antibacterial mechanism

**Fig. 5:**
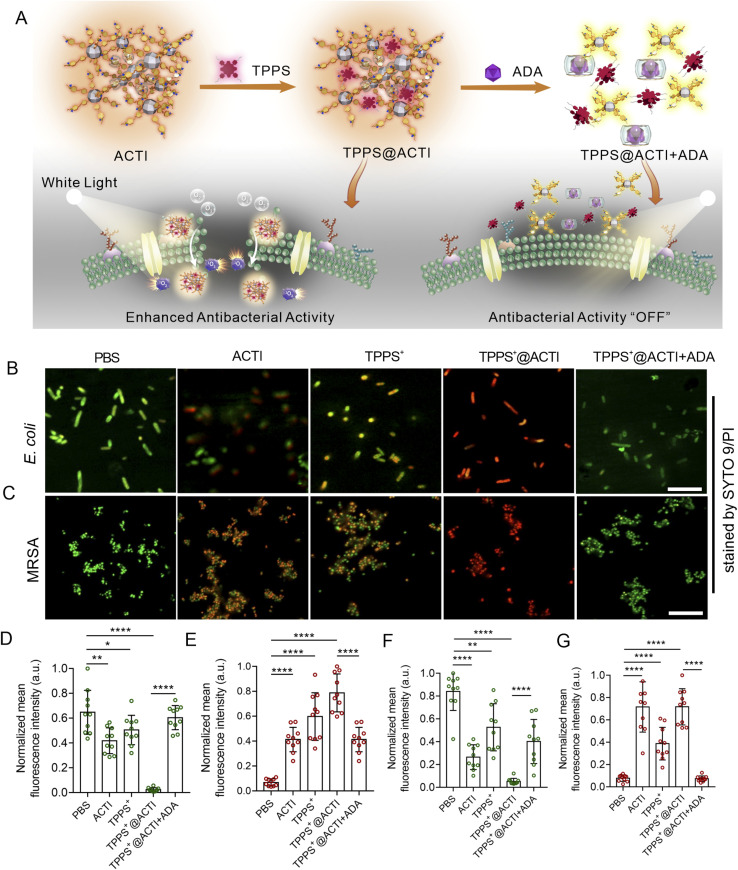
(A) Schematic diagram for the preparation of TPPS@ACTI and its antibacterial mechanism. CLSM images of *E. coli* (B) and MRSA (C) treated with PBS, 10 μM ACTI, TPPS^+^, TPPS^+^@ACTI and TPPS^+^@ACTI + ADA, respectively. Quantification of the fluorescence emission intensity of SYTO 9 (D) and PI (E) from the *E. coli* bacteria and SYTO 9 (F) and PI (G) for MRSA. The normalized mean fluorescence intensity of every image was calculated and presented in bar charts. For each fluorescent dye in each group, the strongest fluorescence emission was set as 1, and other fluorescence signals from the same kind of dye were calculated as a percentage. All groups containing TPPS were illuminated (400–700 nm, 50 mW cm^−2^) and marked as TPPS^+^. Both *E. coli* and MRSA were stained with SYTO 9 (green: live, Ex: 488 nm, and Em: 500–550 nm) and PI (red: dead, Ex: 535 nm, and Em: 590–640 nm). Scale bars are 10 μm. *, ** and *** indicate *P* < 0.05, 0.01 and 0.001 compared to other groups using Student's *t*-test, respectively.

To gain insight into the interaction between ACTI and *E. coli* or MRSA, scanning electron microscopy (SEM) was used to investigate the antibacterial mechanism by observing the morphologies of the bacteria. As shown in Fig. 4G, *E. coli* in the PBS-treated group maintained an integral cell membrane, and the shape and appearance of *E. coli* were also clearly discernible. However, both bacterial cell membranes of *E. coli* treated with TPPS and ACTI upon white light illumination were wrinkled, collapsed and partially merged. In addition, the *E. coli* treated with TPPS@ACTI under white light illumination were significantly damaged. Meanwhile, from the last column of images in Fig. 4G, we can observe that the bacterial morphologies from the group of TPPS@ACTI + ADA upon irradiation remained intact and were not destroyed, indicating that the antibacterial activity can be effectively stopped by the supramolecular disassembly. Besides, the morphological changes in MRSA treated with ACTI and TPPS were similar to those observed with *E. coli* (Fig. 4H). These results suggest that the bacterial biocidal mechanism can be ascribed to a combination of factors: first, the ACTI was adsorbed to the surface of the bacteria, due to the strong affinity between the positively charged polycationic nature of ACTI and negatively charged lipopolysaccharide of bacterial cell walls, which induces membrane disruption, followed by a loss of barrier function of the outer membrane. Thereafter, ^1^O_2_ generated by TPPS under light illumination can more easily diffuse into the bacterial cytoplasm, facilitating the photodynamic anti-bacterial process and leading to enhanced antibacterial activity. However, after adding ADA, the antibacterial process was reduced because of the supramolecular disassembly of TPPS@ACTI. As such, the large molecular weight and the high positive charge density of ACTI breaks down and disassembles into PSPI molecules, which reduces the destructive effect on bacterial membranes. Also, after being disassembled, TPPS is removed from the ACTI assemblies and aggregates in an aqueous environment, resulting in dramatically reduced singlet oxygen efficiency, thus leading to poor photodynamic anti-bacterial activity (Fig. 5A). Additionally, to substantiate the combined antibacterial mechanism of the TPPS@ACTI complex, we measured the changes in zeta potential. As illustrated in Fig. S19, the zeta potential of the TPPS@ACTI complex exhibited a reduction when compared with ACTI alone (from 38.7 to 16.9). Nevertheless, the overall antibacterial efficiency is markedly enhanced based on the *in vitro* antibacterial assays (Fig. 4E and F). This suggests that the antibacterial efficacy is a combined effect of positive charge induced membrane disruption and photosensitizer (TPPS) induced photodynamic inactivation. Despite the decrease in positive charge density (indicated by the zeta potential), the TPPS-mediated photodynamic antibacterial activity results in an overall improvement in antibacterial activity. On the other hand, we observed that the fluorescence of the membrane potential probe DiSC3(5) was enhanced upon the addition of TPPS@ACTI, indicating alterations in bacterial membrane potential and thereby confirming its direct interaction with the bacterial cell membrane (Fig. S20). Moreover, taking advantage of the intrinsic fluorescence of TPPS@ACTI, we found that TPPS@ACTI exhibits fluorescence enhancement in the presence of teichoic acid (TA), a characteristic component of the MRSA cell membrane, further corroborating this specific interaction (Fig. S21). Furthermore, lipid peroxidation analysis using the fluorescent probe BODIPY-C11 revealed oxidative modifications of cell membrane lipids (Fig. S22), suggesting that TPPS@ACTI not only interacts with the bacterial membrane but also induces lipid oxidation upon such interactions. Collectively, these results provide comprehensive mechanistic insights, demonstrating that the enhanced antibacterial activity of TPPS@ACTI originates from the synergistic effects of membrane perturbation, direct membrane interaction, and subsequent photodynamically induced lipid oxidation.

To further explore the antibacterial mechanism, we examined bacterial membrane damage from another perspective by staining using fluorescent dyes, *i.e.* an SYTO 9/PI bacterial viability kit (SYTO 9/PI live/death bacterial staining).^40,41^ Green fluorescence generated from SYTO 9 indicates live bacteria and red fluorescence from PI means that the bacterial cell membrane is compromised or the bacteria are dead. As shown in Fig. 5B, D and E, for *E. coli*, upon treatment with TPPS under irradiation or ACTI alone, the bacterial viability was decreased, when compared with those of the bacteria treated with PBS (blank control group), while the viability of bacteria using TPPS@ACTI upon irradiation decreased dramatically, indicating synergistically enhanced anti-bacterial activity of TPPS@ACTI under white light irradiation. More importantly, the antibacterial activity can be effectively reversed by adding ADA. These results are consistent with the previous CFU reduction results shown in Fig. 4C. Additionally, we also investigated the Gram-positive bacteria MRSA, as shown in Fig. 5C, F and G. TPPS@ACTI upon white light irradiation exhibits similar antibacterial phenomena as with *E. coli*, suggesting that the strategy of using reversible supramolecular antibiotics works effectively for both Gram-negative and Gram-positive bacteria. On the other hand, we examined the interactions of PSPI and ACTI with mammal cancer cells (Hep G2 cells). As shown in Fig. S23, the cell membrane from cells treated with ACTI is destroyed and the boundary is blurred. Moreover, due to the disruption of the cell membrane, the widely applied membrane staining dye (Dil) enters the cell interior along with ACTI. However, PSPI exhibited a weaker interaction with the cell membrane. These phenomena also serve as clear evidence that ACTI with a high positive charge density exerts a stronger disruptive effect on cell membranes.

### 2.5 *In vivo* antibacterial activity

**Fig. 6:**
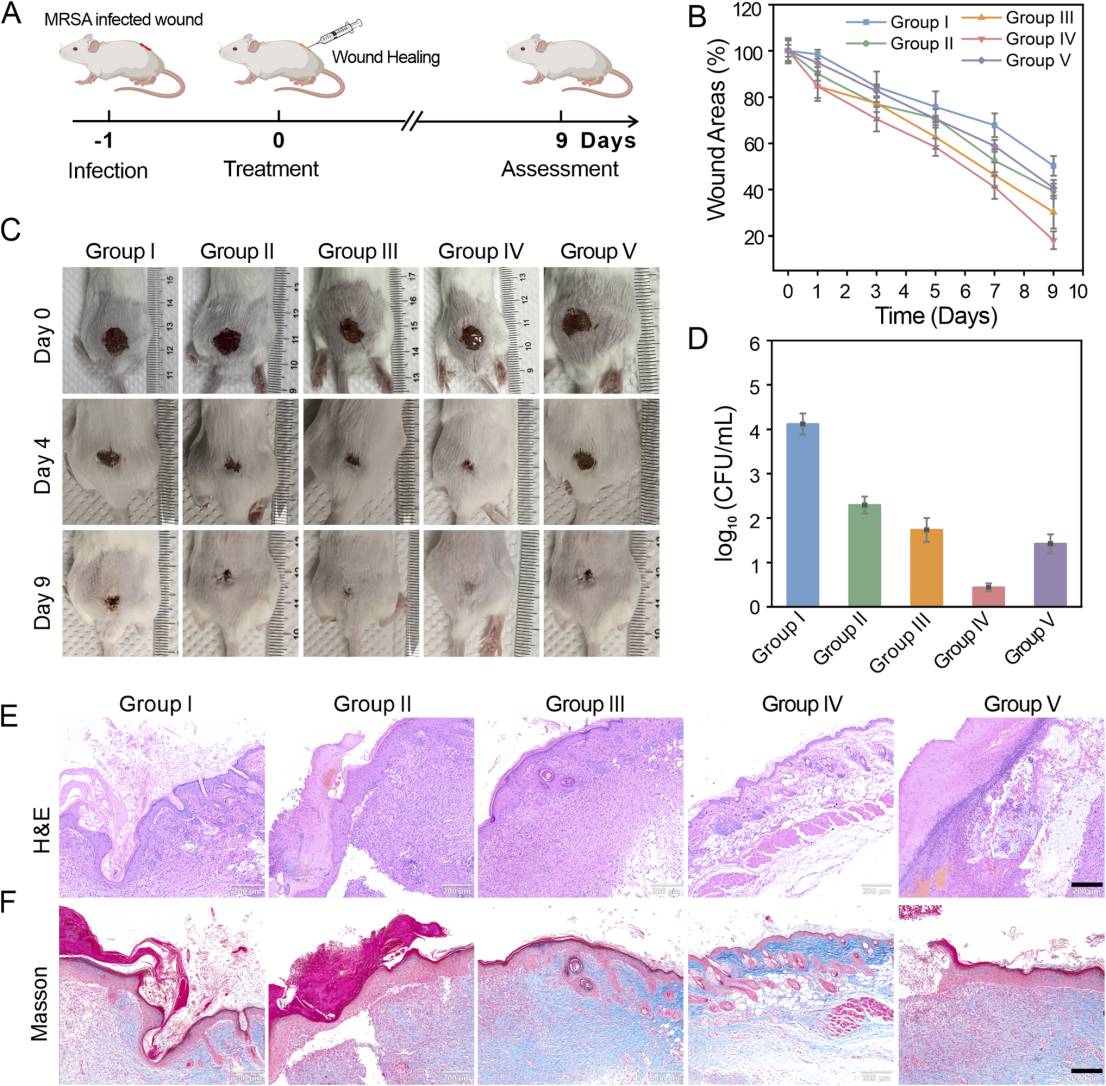
Treatment of MRSA infection with TPPS^+^@ACTI on mouse wounds. (A) The flowchart of the treatment process. (B) Changes in wound areas for MRSA infected mice after various treatments. Group I: saline; Group II: TPPS upon irradiation; Group III: ACTI; Group IV: TPPS^+^@ACTI; Group V: TPPS^+^@ACTI + ADA. (C) Photographs of MRSA infected mice wounds after various treatments. (D) CFU values of MRSA from infected tissues in various treatment groups by using the plate counting method. Photomicrographs of H&E (E) and Masson's trichrome (F) stained sections originated from mice upon different treatments at day 9. [TPPS^+^@ACTI] = 10 μM. Scale bars, 200 μm.

Encouraged by the promising *in vitro* antibacterial efficacy of ACTI, we proceeded to assess its antibacterial activity in a murine model. Prior to assessing the antibacterial efficacy, we tested the cytotoxicity of TPPS@ACTI toward mammalian cells (NIH/3T3 fibroblasts) using a standard MTT assay. As shown in Fig. S24, with increasing concentrations of TPPS@ACTI, its cytotoxicity toward cells increases, due to the higher positive charge density. On the other hand, although TPPS@ACTI exhibited moderate cytotoxicity (about 70% viability) at 10 μM, which may be considered insufficiently safe for healthy cells, the cytotoxicity could be mitigated using a supramolecular disassembly strategy by adding a competing guest (ADA) into the system (resulting in about 85% viability). This observation validates the effectiveness of our strategy from an *in vitro* perspective: switchable supramolecular polycationic assemblies for tunable cell membrane-disrupting capability. To balance the biosafety (illustrated in Fig. S24) and antibacterial efficacy (illustrated in Fig. 4E), a concentration of 10 μM was selected for the *in vivo* antibacterial assays. The *in vivo* antibacterial ability of ACTI was estimated by establishing MRSA infected wounds (Fig. 6A). The MRSA infected mice were divided into five groups based on different treatments: saline (Group I), TPPS under irradiation (Group II), ACTI (Group III), TPPS@ACTI under irradiation (Group IV) and TPPS@ACTI + ADA (Group V). For Group II and Group IV, the infected wounds were irradiated with white light (0.5 W cm^−2^, 5 min) after the antibacterial agents were administrated. To evaluate the antibacterial treatment efficacy, we measured the wound size during the wound healing period, every 2 days. As illustrated in Fig. 6B and C, the areas of infected wounds treated with TPPS@ACTI under irradiation and ACTI have a significant healing effect when compared with the saline treatment group (Group I), reduced to about 20% and 35% of the original wound area, indicating that TPPS@ACTI under irradiation and ACTI treatment have adventitious effects on wound healing. In contrast, TPPS under the irradiation treatment group exhibited only minor wound healing. In addition, the wound areas of mice treated with TPPS@ACTI + ADA are bigger than those of TPPS@ACTI treated mice, indicating that the strategy of regulating antimicrobial activity by adding competitive guest molecules (ADA) to induce ACTI disassembly is effective *in vivo*. In addition, the *in vivo* biocidal efficacy of each group was simultaneously evaluated. As depicted in Fig. 6D, treatment with TPPS under light exposure and ACTI resulted in only modest reductions in viable bacterial counts, by approximately 1.8 and 2.4 log units, respectively. In contrast, the application of TPPS@ACTI combined with light irradiation resulted in a notable bacterial reduction of approximately 3.68 log units, demonstrating a markedly superior antibacterial efficacy against *S. aureus*-infected wounds compared to monotherapy. Moreover, this also further demonstrates that the supramolecular polycations (ACTI) exhibit reversible antibacterial activity.

Histological examination was conducted to evaluate the wound healing efficacy following various treatments.^42^ In Fig. 6E, hematoxylin and eosin (H&E) staining pictures clearly demonstrate that a complete epidermal layer is exclusively present in the TPPS@ACTI group under irradiation, whereas the other treatment groups display increased infiltration of inflammatory cells (saline, TPPS^+^, ACTI, TPPS^+^@ACTI and TPPS^+^@ACTI + ADA). Moreover, the emergence of hair follicles in the TPPS^+^@ACTI group reflects a more advanced stage of tissue repair. Masson's trichrome staining was further utilized to evaluate collagen accumulation within the wound tissues. As illustrated in Fig. 6F, compared to the controls, the skin tissue receiving TPPS@ACTI treatment exhibited denser and more organized collagen fibers, as shown by the blue staining, highlighting improved healing facilitated by the combined effect of ACTI and TPPS-induced photodynamic therapy. Thus, integrating the antibacterial capability of ACTI with TPPS-induced photodynamic inactivation proves to be more effective in promoting the healing of infected wounds.

The *in vivo* safety evaluation of ACTI was also determined. No obvious weight loss or other abnormal behaviours were observed after the mice were treated with ACTI (Fig. S25). A comprehensive histological analysis using H&E staining was conducted to gain further insight into the systemic toxicity of ACTI *in vivo*. The main organs (heart, liver, spleen, lung, and kidney) from all five experimental groups were excised and subjected to staining. According to Fig. S26, no discernible pathological changes or inflammatory responses were detected in the major organs of the ACTI-treated mice.

## 3 Conclusion

In conclusion, we have successfully developed a pyridinium-based supramolecular assembly that functions as a reversible supramolecular antibiotic, offering controlled antibacterial activity. The as-formed Adaptive Cationic Therapeutic Integrated (ACTI) system with high-density cationic charges can effectively inactivate both Gram-negative (*E. coli*) and drug resistance Gram-positive bacteria (MRSA) by disrupting their cell membranes. This disruption is a form of physical damage, which drug-resistant bacteria cannot effectively counteract. Significantly, by introducing a competitive guest molecule, ADA, the membrane-disrupting and antibacterial activities of ACTI can be effectively turned “OFF” *via* supramolecular disassembly. This process prevents prolonged membrane damage, enhances biosafety, and enables precise, on-demand regulation of antibacterial activity. While our focus has primarily been on antibacterial efficacy, it is expected that these intelligent, fluorescence-adjustable polycationic networks hold potential for applications in diverse areas such as drug delivery, nucleic acid delivery and optoelectronic technologies.

## Ethical statement

All *in vivo* animal experiments were approved by the Experimental Animal Ethics Committee of Shandong First Medical University (no. W202302270103).

## Author contributions

Jia Chen, Xueqian Wang and Xue Wang performed the organic synthesis, characterized the photophysical properties and performed titrations; Jia Chen and Mengrui Zhang collected the data, did the analysis and drew the scientific figures; Ran Wang, Xinxing Lyu and Yunjian Xu performed the antibacterial assays and investigated the antibacterial mechanism; Xueqian Wang performed cell experiments and investigated the cytotoxicity on NIH/3T3 fibroblasts. Jia Chen, Luling Wu and Xintian Shao wrote the original manuscript; Jia Chen and Xintian Shao developed the project and obtained funding. Tony D. James provided supervision and edited the manuscript.

## Conflicts of interest

The authors declare no conflicts of interest.

## Supplementary Material

SC-016-D5SC05035A-s001

## Data Availability

Any other data that support the findings of this work are available from the corresponding author upon reasonable request. All data of this work are provided in the article and as part of the supplementary information (SI). See DOI: https://doi.org/10.1039/d5sc05035a.
