# Supplementary material for: Switchable supramolecular polycationic assemblies for tunable antibacterial strategies against antibiotic resistance
Source: Chem Sci. 2025 Oct 7. Online ahead of print. doi: 10.1039/d5sc05035a (PMC12502198; doi:10.1039/d5sc05035a)
Supplement: Supplementary file 1 [file SC-OLF-D5SC05035A-s001.pdf]

Supplementary Information (SI) for Chemical Science.
This journal is © The Royal Society of Chemistry 2025

Supporting Information

Switchable Supramolecular Polycationic Assemblies for Tunable Antibacterial Strategies Against Antibiotic Resistance

Jia Chen,[ab]* Xueqian Wang,[b] Mengrui Zhang,[b] Xue Wang,[ab] Ran Wang,[b] Xinxing Lyu,[b] Yunjian Xu,[a] Xintian Shao,[b]* Luling Wu[cd]* and Tony D. James[d]

[a]School of Radiology, Shandong First Medical University & Shandong Academy of Medical Sciences, Tai'an, Shandong 271016, China.
[b]Medical Science and Technology Innovation Center, Shandong First Medical University & Shandong Academy of Medical Sciences, Jinan, Shandong 250117, China.
[c]State Key Laboratory of Analytical Chemistry for Life Science, School of Chemistry and Chemical Engineering, Nanjing University, 163 Xianlin Avenue, Nanjing, 210023, China
[d]Department of Chemistry, University of Bath, Bath BA2 7AY UK

E-mail: chenjia@sdfmu.edu.cn; wllcyl@126.com; shaoxintian@sdfmu.edu.cn

Table of Contents

1. Reagents and Chemicals
2. Instrumentation
3. ITC Assays
4. Preparation of Bacterial Solutions
5. Antibacterial Activity and Reversing the Antibacterial Activity
6. Determination of the Loading Efficiency of TPPS on TPPS@ACTI assemblies
7. Photodynamic anti-bacterial activity of TPPS@ACTI
8. Bacterial Morphology Study
9. Zeta Potential Measurements
10. Bacterial Live/Dead Staining
11. Confocal Laser Scanning Microscopy (CLSM) Imaging
12. Statistical Analysis
13. Animal Experiments
14. Synthetic Scheme and Details
15. Related Figures
16. References

1、Reagents and Chemicals

Octvinyl-POSS was purchased from Hybrid Plastics company (USA), the singlet oxygen trapper in aqueous solutions, 9,10-Anthracenediyl-bis(methylene)dimalonic Acid (ABDA), was purchased from Sigma-Aldrich. The in vitro singlet oxygen detection kit (DCFH-DA) was obtained from Beyotime biotechnology, China. The LIVE/DEAD BacLight Bacterial Viability Kit (for microscopy and quantitative assays), Cat. No. L7012 (Syto 9/PI), was purchased from ThermoFisher Scientific. The cucurbituril[8] (CB[8]) compound was obtained as a gift from the Ruibing Wang Group at the University of Macao. The other chemicals and reagents are obtained from Sinopharm Chemical Reagent Co., Ltd without further purification. The organic solvent used in this project are purified and anhydrous. All the reactions are executed at an inert gas atmosphere.

2、Instrumentation

The NMR spectra are obtained on Bruker 600 MHz ASCENDTM. Mass spectra are collected from SCIEX X500R QTOF mass system. We record UV-Vis absorption signals on HACH DR6000 spectrophotometer in 10 mm path quartz cell. Photoluminescence spectra of samples are measured on Horiba fluoroMax-4 spectrofluorometer. TEM characterization was performed on a Hitachi HT7700 microscope operating at 100 kV accelerating voltage and SEM images are acquired on a Hitachi Regulus 8100 operating at 8.0 kV, and the magnification is 12K times. The CLSM images were obtained on the machine of Zeiss LSM 900/980 with airyscan. We acquired the hydrodynamic sizes distribution on Zetasizer Nano ZS, Malvern Instruments Inc. The white light applied for the photodynamic antibacterial assay is generated from OSL2 High-Intensity Fiber Light Source, Thorlabs.

3、ITC Assays

0.3 mM SPI and 20 μM CB[8] were individually dissolved in ultrapure water and diluted into specified concentrations. The SPI was set into the sample cell, and the CB[8] was placed in syringe. All the solutions were sonicated for degassing before ITC determinations. The ITC titrations were performed by titrating 19 drops of CB[8] into the solution of SPI, and the heat evolution was recorded. The heat generated from the dilution of CB[8] was revised by titrating with ultrapure water and the data was subtracted from those of the host guest titrations. All ITC assays were conducted three times at 25°C, and the data were analyzed with the built-in software of MicroCal PEAQ ITC Analysis instrument.

4、Preparation of Bacterial Solutions

Both the *E. coli* (ATCC 25922) and methicillin-resistant *Staphylococcus aureus* (MRSA, ATCC 33592) came from respective solid lysogeny broth (LB, 5 $g \cdot L^{-1}$ yeast extract, 10 $g \cdot L^{-1}$ NaCl, and 10 $g \cdot L^{-1}$ tryptone) agar plates. Then, they were added to liquid LB culture medium and cultured at 37 °C with shaking. After incubation for 24 h, bacteria were collected through centrifuging (7,200 g for 5 mins) and washed with PBS (1 mM, pH = 7.4) twice. The obtained bacterial pellets were re-suspended in PBS and diluted to A_{600} = 0.01 (10^6 ~10^7 bacteria/mL) for later experiments, the bacterial concentrations were determined by testing their optical densities at 600 nm.

5 Antibacterial Activity and Reversing the Antibacterial Activity

For the ACTI antibacterial activity, various concentrations (2.5, 5, 10, 20 and 40 μM) of ACTI were incubated with bacteria (*E. coli* or MRSA) for 30 mins in the dark. For the reversible biocidal activity investigation, 20 equivalents of ADA were added after ACTI and TPPS@SAMB was administrated into the bacterial culture medium, respectively. After the treatment with ACTI, the bacteria were harvested, washed and diluted with PBS buffer. The bacteria dilutions were then spread onto a solid LB agar plate. The CFU numbers were determined by counting the bacterial colonies formed after 18h incubation. Each group was performed three times. And the CFU reduction can be calculated as:

$$CFU\ Reduction = 1 - \frac{number\ of\ CFU\ (treated)}{nmuber\ of\ CFU\ (Control)}$$

6 Determination of the Loading Efficiency of TPPS on TPPS@ACTI assemblies

ACTI was positively charged, TPPS with equal molar negative charges as ACTI was added into the ACTI assemblies. 0.03 mL of 40 mM TPPS (excess TPPS) was added into 10 mL of 0.1 mM ACTI aqueous solution under ultrasonification for 10 mins and stirring for 10 mins at room temperature. Then the mixture was purified with high-speed centrifugation at 12,000 rpm for 15 mins to collect the precipitate. By measuring the absorption of TPPS in the supernatant diluted 10 fold, the unloaded TPPS could be calculated (concentration: 55.9 μM) according to the standard curve of TPPS (Figure S17). The loading content of TPPS onto ACTI can be determined by subtraction TPPS in supernatant from the total added (Figure S18). Finally, the loading efficiency of TPPS on TPPS@ACTI assemblies can be calculated to be 64.1%, meaning about 10 μmol TPPS@ACTI contains 6.41 μmol TPPS.

7 Photodynamic anti-bacterial activity of TPPS@ACTI

For the TPPS@ACTI antibacterial activity, 10 μM TPPS@ACTI were incubated with bacteria (*E. coli* or MRSA) for 30 mins in the dark. White light illumination was chosen to perform photodynamic anti-bacterial activity as white light (400-700 nm) can completely cover the B-band and Q-band absorption of TPPS and is convenient to access. The mixtures of bacteria and TPPS@ACTI irradiated for a set time. The illumination power (24 J/cm^2: 50 mW/cm^2, 8 mins) applied is comparable to white-light photodynamic anti-bacterial activity reported in previous literature.[1] After treatment, the bacteria were harvested, washed and diluted with PBS buffer. The following procedures are the same as described the antibacterial activity of ACTI in the dark.

8 Bacterial Morphology Study

After the treatment of bacteria (*E. coli* or MRSA) with ACTI, TPPS and TPPS@ACTI in the presence or absence of white light irradiation, the bacteria were swiftly stabilized using 2.5% glutaraldehyde for 4 hours at 4°C. This was followed by a step-by-step dehydration process using 20%, 50%, 80%, and 100% ethanol, each for a duration of 10 mins. The bacterial morphology study was performed on a Hitachi Regulus 8100 operating at 8.0 kV, and the magnification is 12K times. Before undergoing SEM analysis, the samples received a gold coating for 160s.

9 Zeta Potential Measurements

The bacteria in PBS were incubated with PSPI, PSPI/CB[8] and CB[8] at 37°C for 10 min. After that, the unbound PSPI, PSPI/CB[8] and CB[8] was removed by centrifugation at 7,200 g for 5 mins. The obtained pellets were washed with water, after centrifugation at 7,200 g for 5 mins, then re-suspended in 1.5 mL PBS and kept on ice. The specimens were prepared for zeta potential measurements. The *E. coli* incubated without PSPI and PSPI/CB[8] as the control group was conducted under the same condition.

10 Bacterial Live/Dead Staining

The bacterial Live/Dead staining assays were performed by using a commercial kit, LIVE/DEAD BacLight bacterial viability kit (Cat. No. L7012), from ThermoFisher Scientific. This kit is used to assess the viability of bacterial populations as a function of the membrane integrity of the cell. The mechanism is that Cells with a compromised membrane that are considered to be dead or dying will stain red, whereas cells with an intact membrane will stain green.

Bacteria (*E. coli* or MRSA) were grown and harvested, washed, and re-suspended in PBS (pH = 7.4) to an OD_{600} = 0.3 bacterial solution. Separated bacterial solutions were treated by respective ACTI, TPPS and TPPS@ACTI for an allowed time slot. After the process of antibacterial activity, the bacteria were centrifugated at 7,200 g for 5 mins, then washed by a solution of 500 mM NaCl to thoroughly remove these fluorescent substance (PSPI, ACTI and TPPS@ACTI). At last, the harvested pellets were re-dispersed with PBS. The resulting samples were stained with SYTO 9 and PI according to the manufactures protocol (1 μM Syto 9 and 1 μM PI for incubating 30 mins). Afterwards the bacteria were centrifugated and the supernatant was carefully removed, and the stained bacteria were transferred onto a glass microscope slide for further CLSM image collection.

11 Confocal Laser Scanning Microscopy (CLSM) Imaging

The bacteria (*E. coli* and MRSA) were incubated with specific samples at 37 °C for 30 mins. After discarding the unbound samples in media by centrifugation (7,200 g for 5 mins), the residue was re-suspended in PBS and kept on ice. 8-10 μL aliquots of suspensions were added to clean glass slides, and then the coverslips were slightly covered. The specimens were observed under confocal laser scanning microscopy. The control group was bacteria incubated without samples. Different fluorescent emission channels were specifically indicated in CLSM images.

12 Statistical Analysis

In this study, three separate tests were conducted to determine average values, which were presented as mean ± standard deviation. The differences between the untreated control group and the irradiated groups were statistically analyzed by using one-way ANOVA. A p-value of less than 0.05 was deemed significant. *, ** and *** indicate $P < 0.05$, 0.01 and 0.001 comparing to other groups using Student's t-test, respectively.

13 Animal Experiments

All in vivo animal experiments were approved by the Experimental Animal Ethics Committee of Shandong First Medical University (No. W202302270103). A total of 30 female BALB/c mice (6-week ages, about 20 g body weight) were randomly divided into 5 groups, with 6 mice in each group. Group A: Saline; Group B: TPPS upon irradiation; Group C: ACTI; Group D: TPPS@ACTI upon irradiation; Group E: TPPS@ACTI+ADA. The wound on each mouse was inflicted to form a circular shape with a diameter of approximately 10 mm, and infected by MRSA for 12h. The Concentrations of TPPS, ACTI and TPPS@ACTI applied on animal

experiments were set as 64.1 μM, 100 μM, and 100 μM, respectively. The irradiation applied were executed with white light (400-700 nm) at the power density of 0.5 W/cm^2 for 5 mins. For the biosafety evaluation, the body weights of mice were recorded every 2 days, and the major organs (heart, liver, spleen, lung, and kidney) of the mice were collected for histological analysis *via* H&E staining at Day 14 after different treatments. All the slices were observed and captured by using an Olympus VS200 microscope.

14 Synthetic Scheme and Details

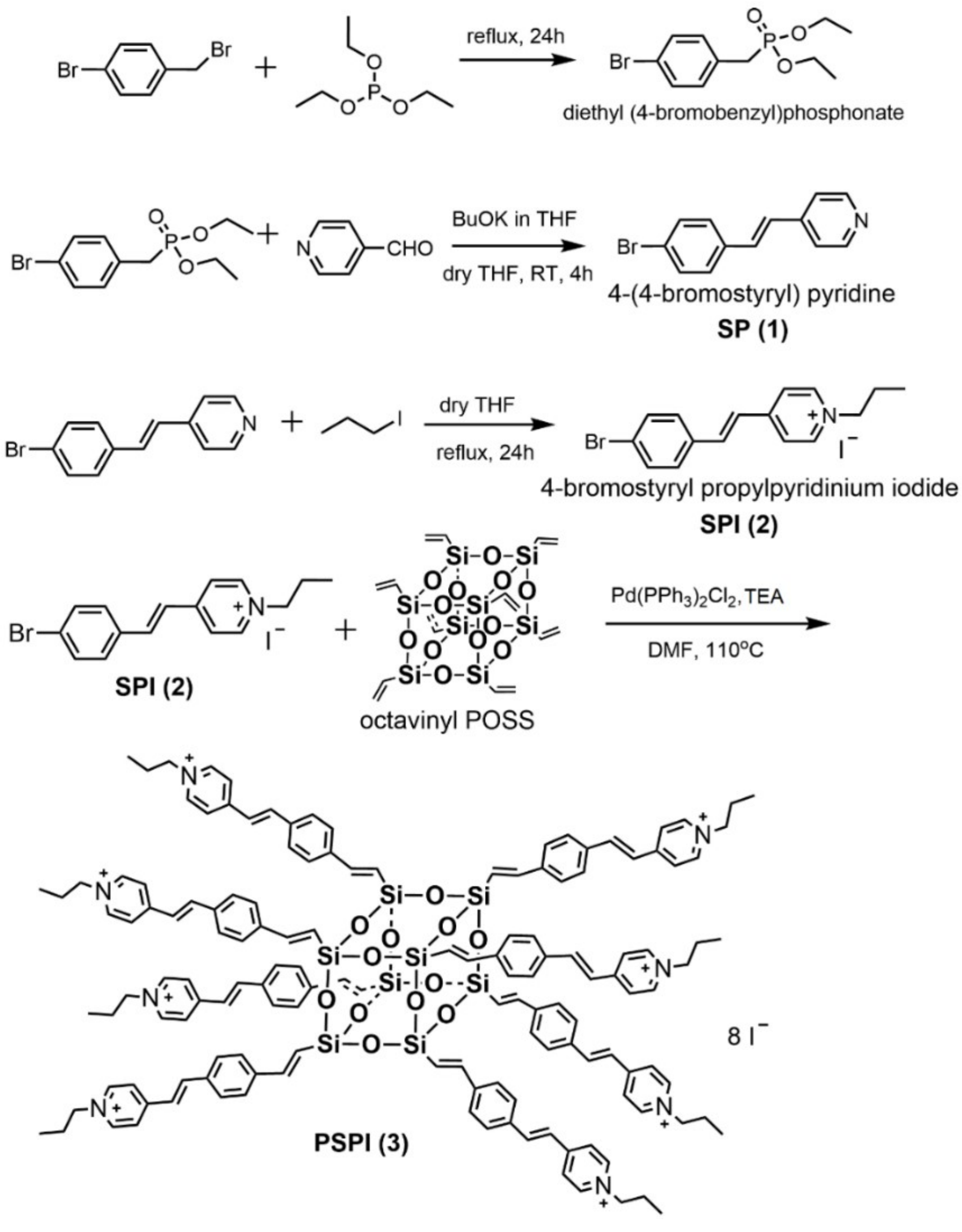

Scheme S1. The synthetic route for PSPI.

Synthesis of diethyl 4-bromobenzylphosphonate. The procedure towards diethyl 4-bromobenzylphosphonate has been reported previously.[2] Briefly, 5 g (20 mmol) 4-bromobenzyl bromide was dissolved in an excess amount of triethylphosphite (about 10 mL,

57.9 mmol). The solution was refluxed for 24 h and then the mixture was cooled to room temperature and the solvent was removed under reduced pressure using a rotatory evaporator. The residue was then purified by flash column chromatography (Hex: EA = 10:1) affording the desired product as a colorless liquid, 5.651 g with a 92% yield. ^{1}H NMR (300 MHz; $CDCl_3$; Me_4Si): 7.40 (d, J = 8.0 Hz, 2H), 7.14 (d, J = 8.0 Hz, 2H), 4.04-3.94 (m, 4H), 3.10 (d, J = 21.7 Hz, 2H), 1.22 (t, J = 7.1 Hz, 6H). ^{13}C NMR (201 MHz; $CDCl_3$; Me_4Si): 131.75 (d, J = 2.8 Hz), 131.54 (d, J = 7.2 Hz), 130.87 (d, J = 8.9 Hz), 121.03 (d, J = 4.5 Hz), 62.33 (d, J = 7.1 Hz), 33.37 (d, J = 139.0 Hz), 16.49 (d, J = 5.9 Hz). HRMS (ESI$^+$): calculated [M+H]$^+$, 307.0093 *m*/*z*, found 306.9918 *m*/*z*. M represents $C_{11}H_{16}BrO_3P$ (chemical formula of compound diethyl 4-bromobenzylphosphonate).

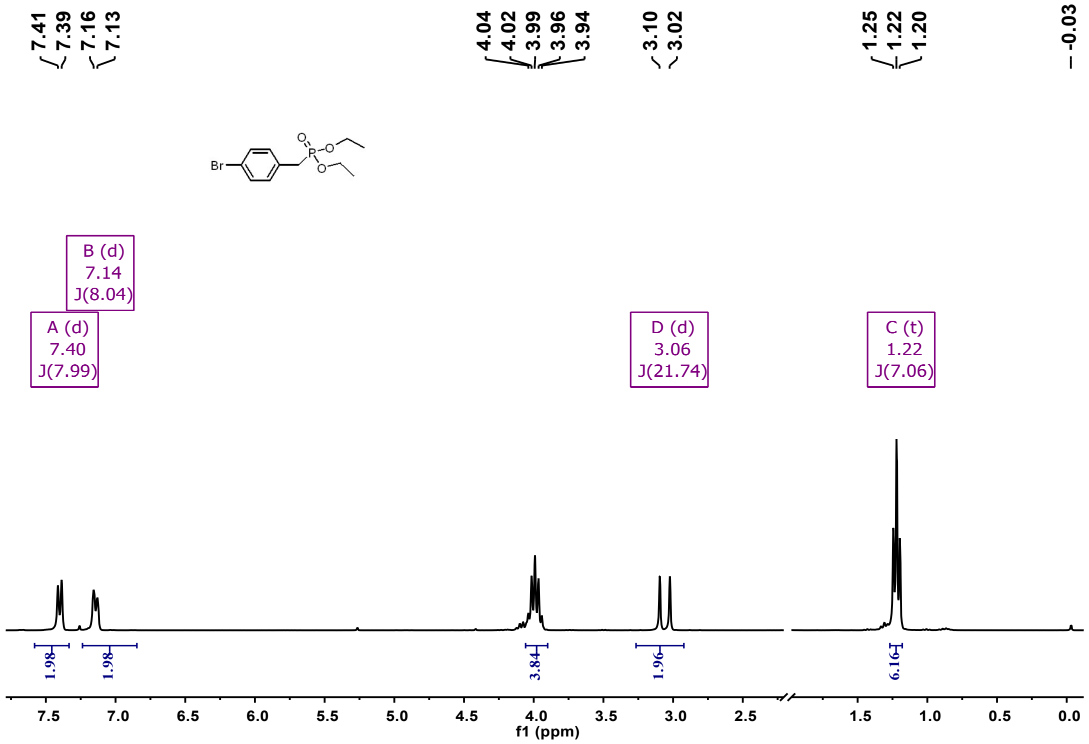

Figure S1. The ^{1}H NMR spectrum of 4-bromobenzylphosphonate.

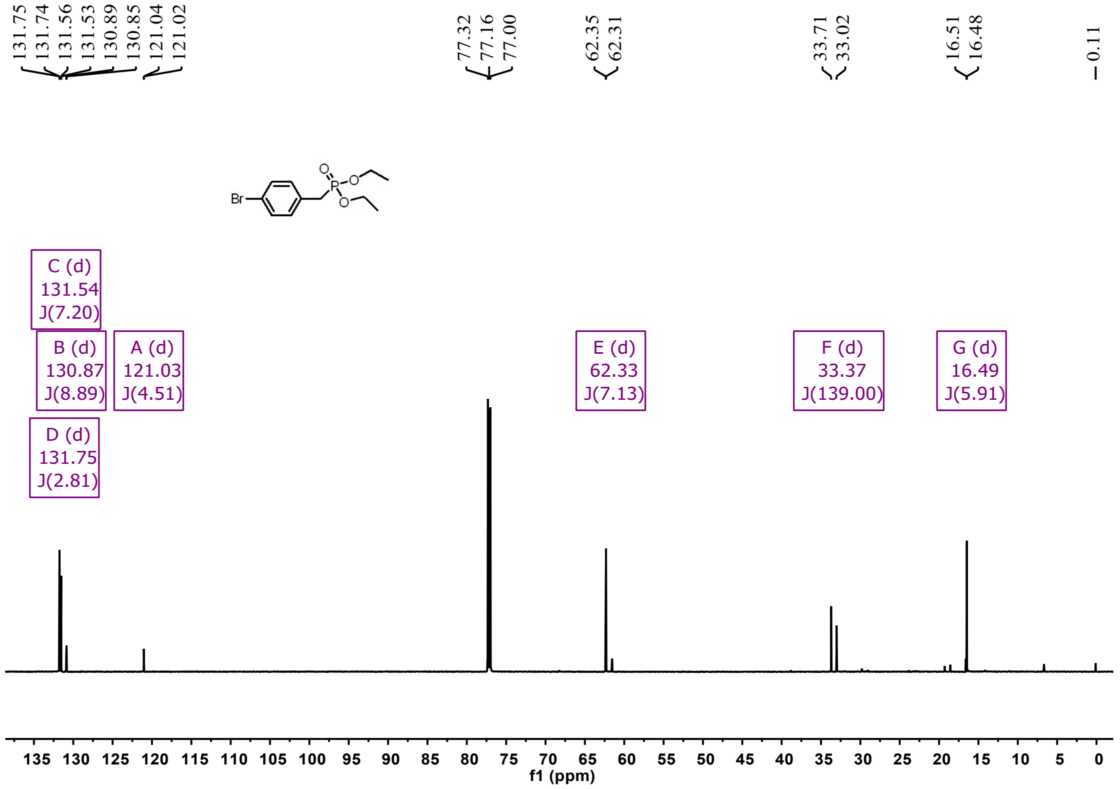

Figure S2. The ^{13}C NMR spectrum of 4-bromobenzylphosphonate.

Synthesis of 4-(4-bromostyryl) pyridine (1). 0.75 g 4-pyridinecarbaldehyde (7 mmol, 1 equiv.) and 2.37g diethyl 4-bromobenzylphosphonate (7.7 mmol, 1.1 equiv.) were added into a N_2 atmosphere protected Schlenk flask, followed by 30 mL anhydrous THF that was injected via a syringe to dissolve all of the reactants. Then about 7.7 mL 1M *t*-BuOK THF solution (7.7 mmol, 1.1 equiv.) was added dropwise into the solution over15 mins. The mixture was then stirred for 4h at room temperature (RT). After which the reaction was poured into 100 mL water, and the precipitate was collected and purified using flash column chromatography affording the desired product as a white powder, 1.548 g in a 85% yield. ^1H NMR (600 MHz; $CDCl_3$; Me_4Si): 8.56 (d, J = 5.4 Hz, 2H), 7.48 (d, J = 8.4 Hz, 2H), 7.36 (d, J = 8.3 Hz, 2H), 7.32 (d, J = 6.1 Hz, 2H), 7.19 (d, J = 16.3 Hz, 1H), 6.96 (d, J = 16.3 Hz, 1H). ^{13}C NMR (151 MHz; $CDCl_3$; Me_4Si): 150.32, 144.27, 135.13, 132.06, 131.90, 128.51, 126.75, 122.73, 120.92. HRMS (ESI$^+$): calculated $[M+H]^+$: 260.0069 *m*/*z*, found 259.9922 *m*/*z*. M represents $C_{13}H_{10}BrN$ (chemical formula of compound **1**).

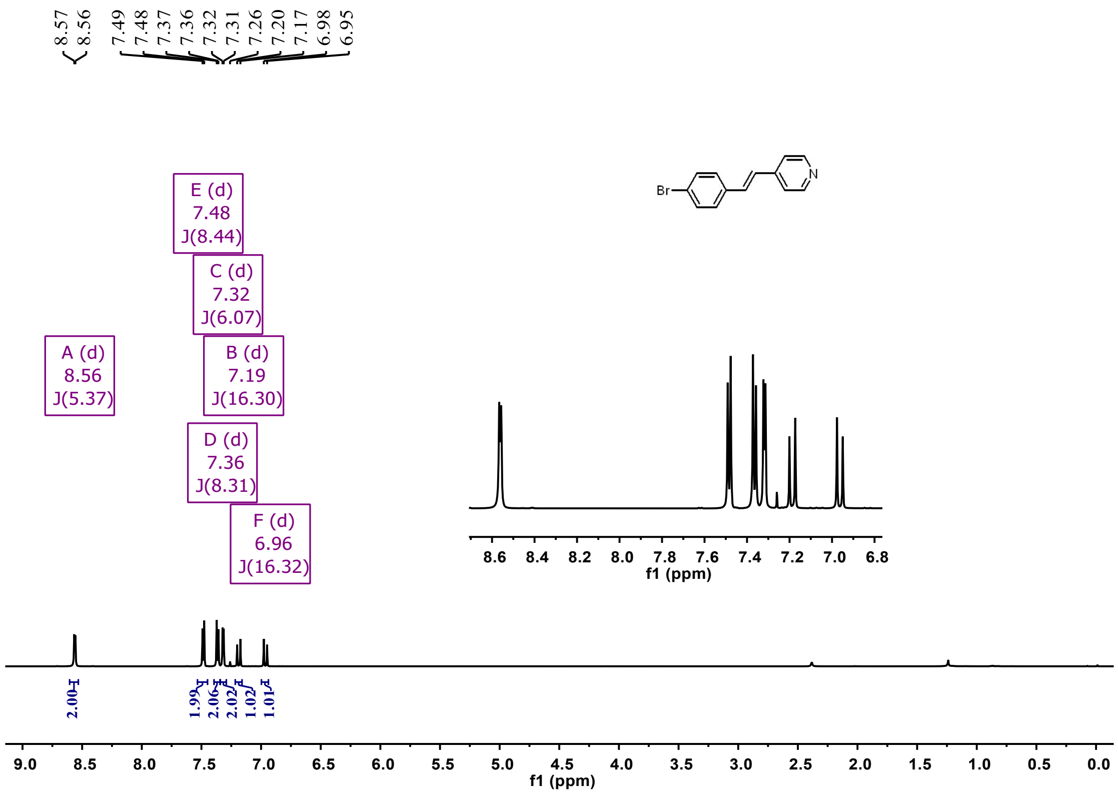

Figure S3. The ^{1}H NMR spectrum of 4-(4-bromostyryl) pyridine.

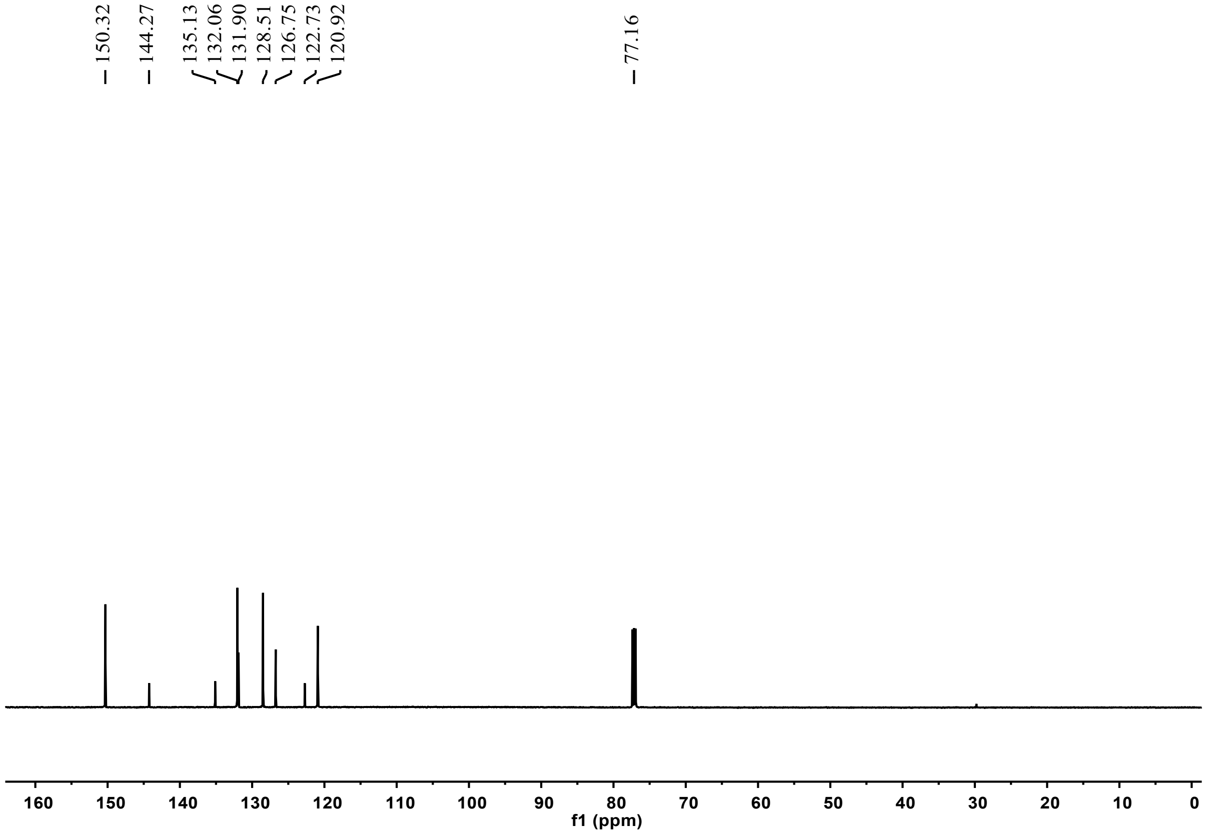

Figure S4. The ^{13}C NMR spectrum of 4-(4-bromostyryl) pyridine.

Synthesis of 4-bromostyryl-1-propylpyridium iodide (SPI, 2). To a solution of 0.56 g 4-(4-bromostyryl) pyridine (2.15 mmol, 1 equiv.) dissolved in 80 mL THF, 731.9 mg iodopropane

(4.3 mmol, 2 equiv.) was added. The mixture was then purged by N_2, then and the reaction was refluxed for 24 h. The reaction was then cooled to room temperature and the precipitate was filtered. The remaining solvent was removed under reduced pressure by a rotary evaporator. The residue and the precipitate we combined and washed with acetonitrile and diethyl ether to provide the desired product in an 89% yield, 0.823 g. ^1H NMR (600 MHz; DMSO-d_6; Me_4Si; Figure S5): 8.97 (d, J = 6.9 Hz, 2H), 8.25 (d, J = 6.9 Hz, 2H), 8.00 (d, J = 16.3 Hz, 1H), 7.73-7.69 (m, 4H), 7.57 (d, J = 16.4 Hz, 1H), 4.47 (t, J = 7.3 Hz, 2H), 1.97-1.91 (m, 2H), 0.90 (t, J = 7.4 Hz, 3H). ^{13}C NMR (151 MHz; DMSO-d_6; Me_4Si; Figure S6): 152.59, 144.41, 139.44, 134.41, 132.17, 129.95, 124.14, 124.03, 123.76, 61.18, 23.96, 10.26. HRMS (ESI$^+$): calculated $[M]^+$ 302.0539 *m*/*z*, found 302.0365 *m*/*z*. M represents $C_{16}H_{17}BrN$ (chemical formula of compound 2).

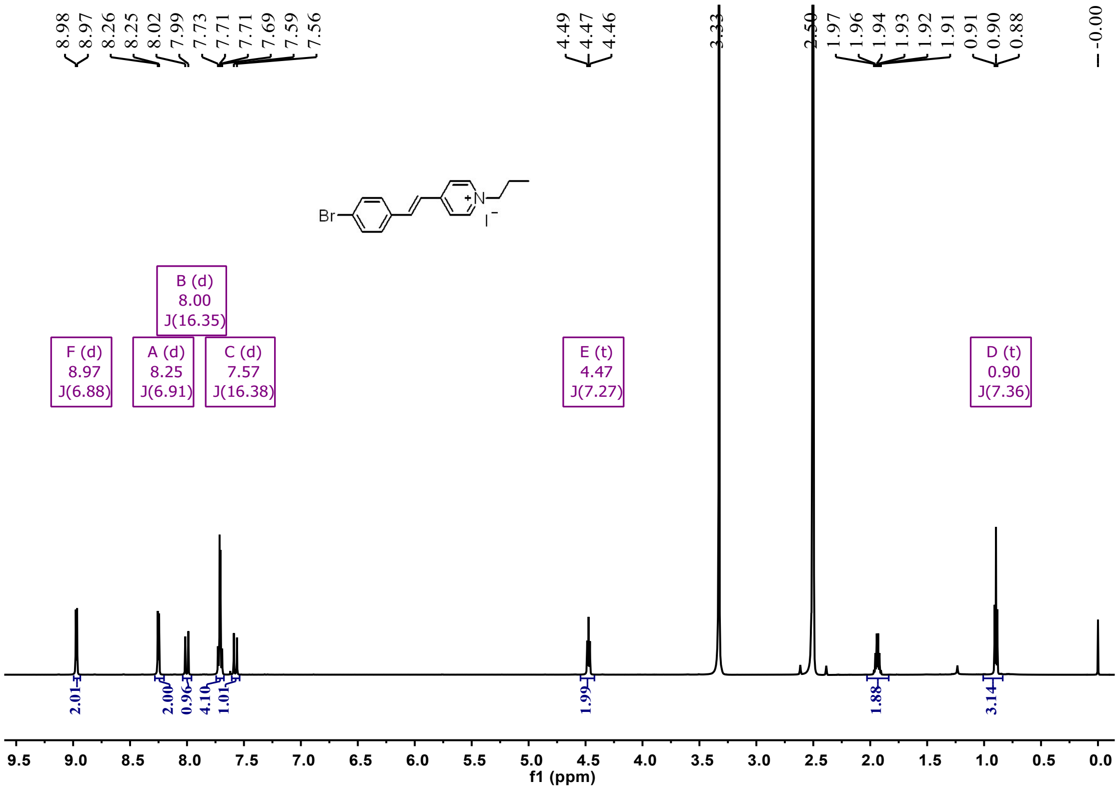

Figure S5. The ^1H NMR spectrum of SPI.

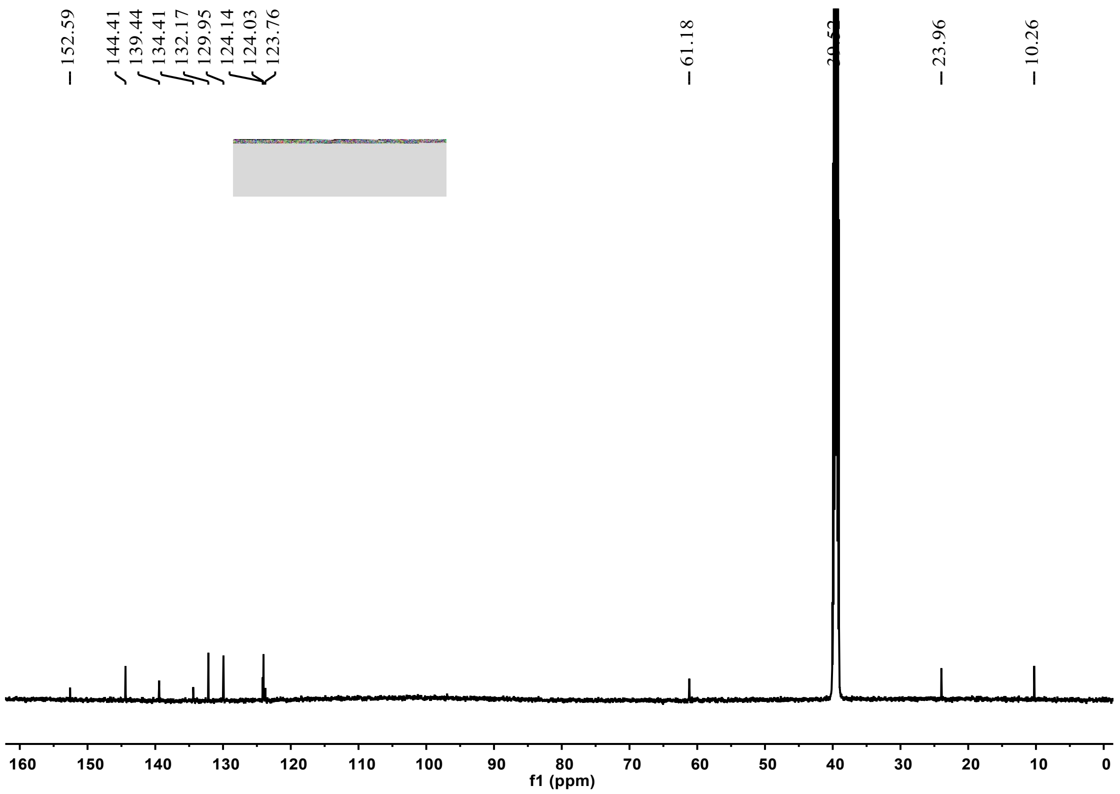

Figure S6. The ^{13}C NMR spectrum of SPI.

Synthesis of PSPI (3). The synthesis of **PSPI** was referenced from previous literatures. [3, 4] To a Schlenk flask, 0. 271 g Octavinyl POSS (0.43 mmol, 1 equiv.), 1.591 g **SPI** (**2**, 3.698 mmol, 8.6 equiv.), 0.435 g triethylamine (4.3 mmol, 10 equiv.) and 51.9 mg $Pd(PPh_3)_2Cl_2$ (0.074 mmol, 8.6*2% equiv.) were placed under argon inert atmosphere. After three cycles of argon gas replacement, anhydrous DMF was injected into the flask, then the mixture was stirred at 110°C for 36h. After cooling to room temperature, it was filtered to remove the catalyst. The filtrate was added to 50 mL acetone, and the yellow precipitate was collected, then washed with diethyl ether. After drying, the final product was obtained as a orange solid, 0.707 g in 48% yield. Due to the oligomer-like properties of PSPI, the ^{1}H NMR signals in the aromatic ring region of PSPI are very crowded and difficult to identify and integrate one by one. ^{1}H NMR (600 MHz; DMSO-d$_6$; Me_4Si; Figure S7 and S8), ^{13}C NMR (150 MHz; DMSO-d_6; Me_4Si; Figure S9). To the characterization of exact mass, it was performed on machine of SCIEX X500R QTOF (mass range isolating quad 1:5 - 2250), and the test method referred a literature on POSS-based ammonium salt.[5] HRMS (ESI$^+$) m/z: calcd for $[M-8I]^{8+}$ 301.3710 *m*/*z*, found 301.1381 *m*/*z*. M represents $C_{144}H_{152}N_8Si_8O_{12}I_8$ (chemical formula of compound **PSPI**).

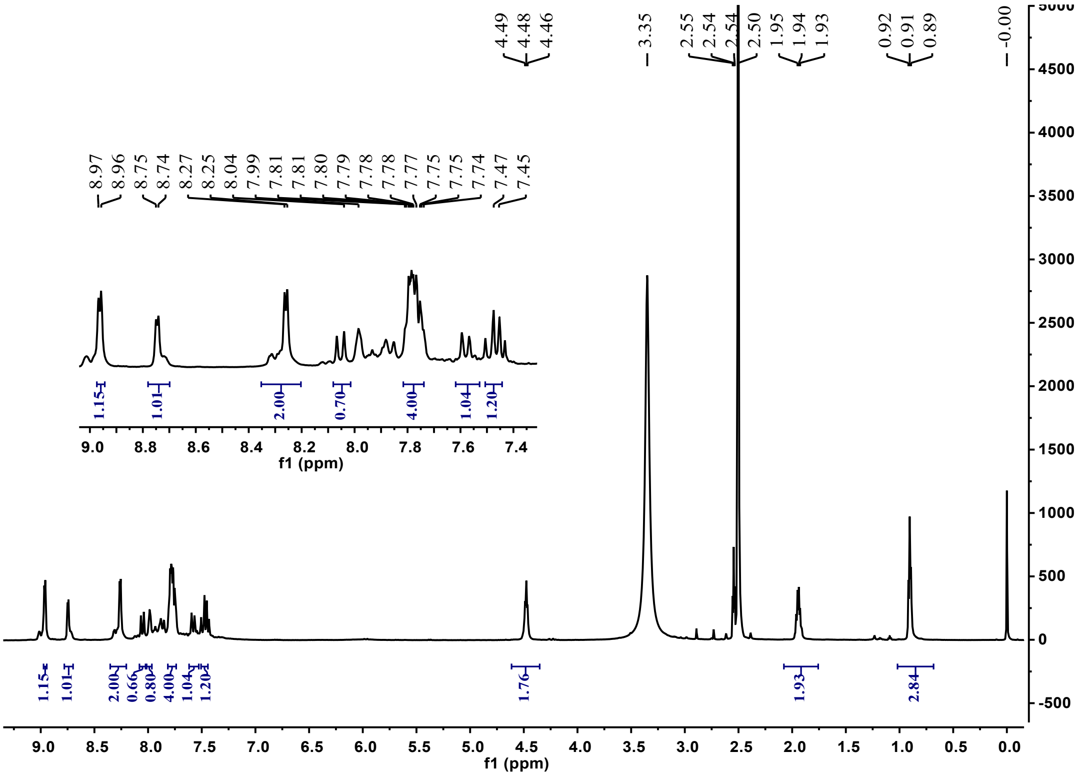

Figure S7. The ^{1}H NMR spectrum of PSPI.

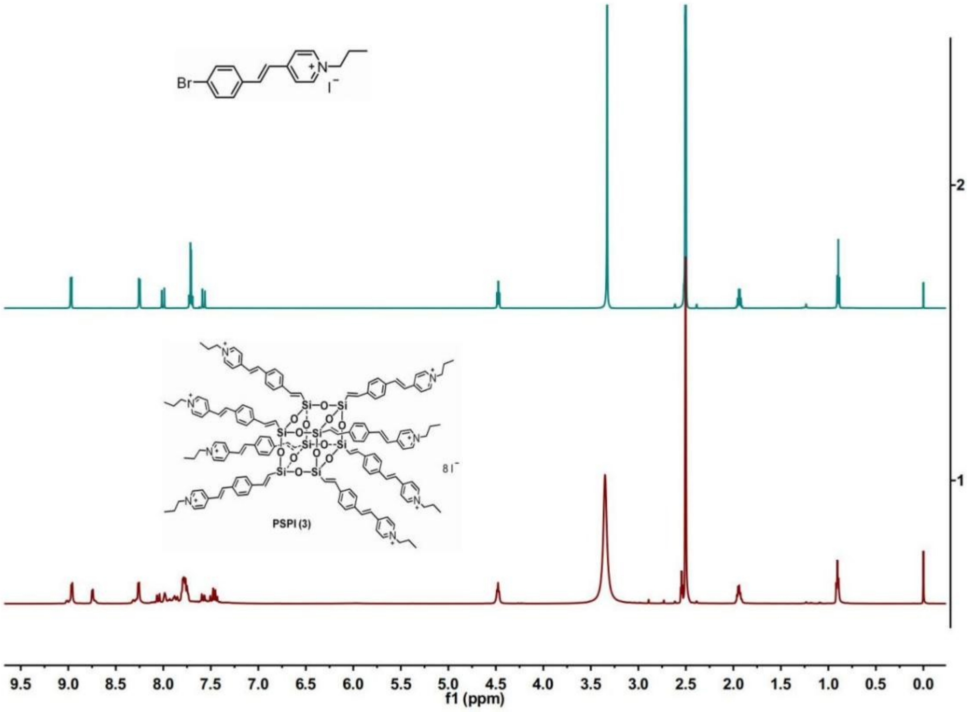

Figure S8. The ^{1}H NMR spectra of SPI (above) and PSPI (below), respectively. The two images are put together for clear comparison.

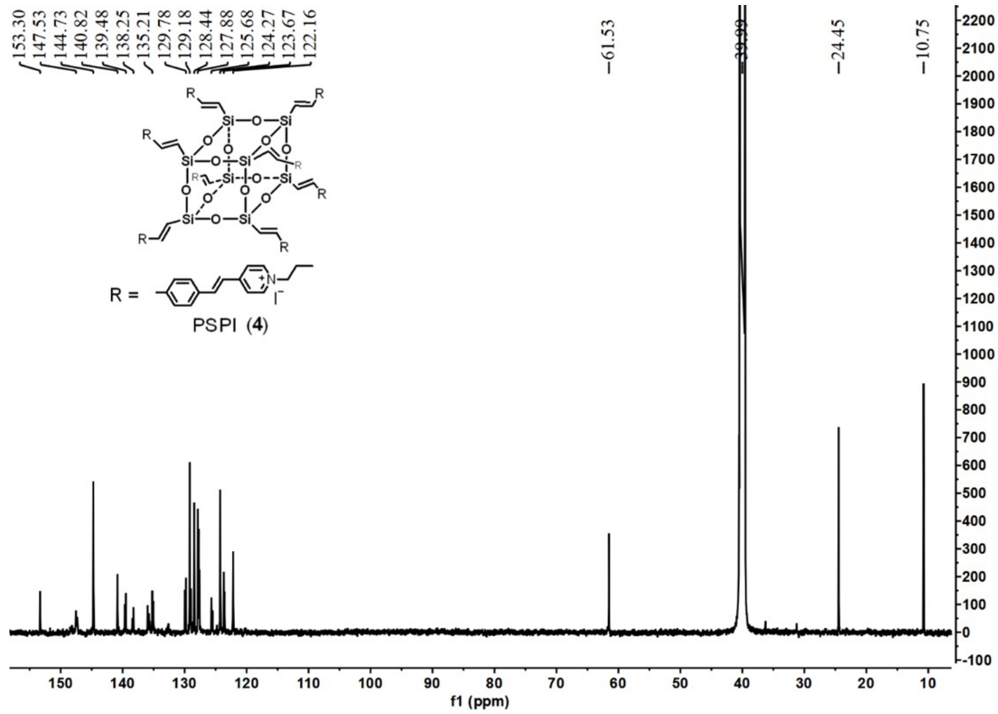

Figure S9. The ^{13}C NMR spectrum of PSPI.

15 Related Figures

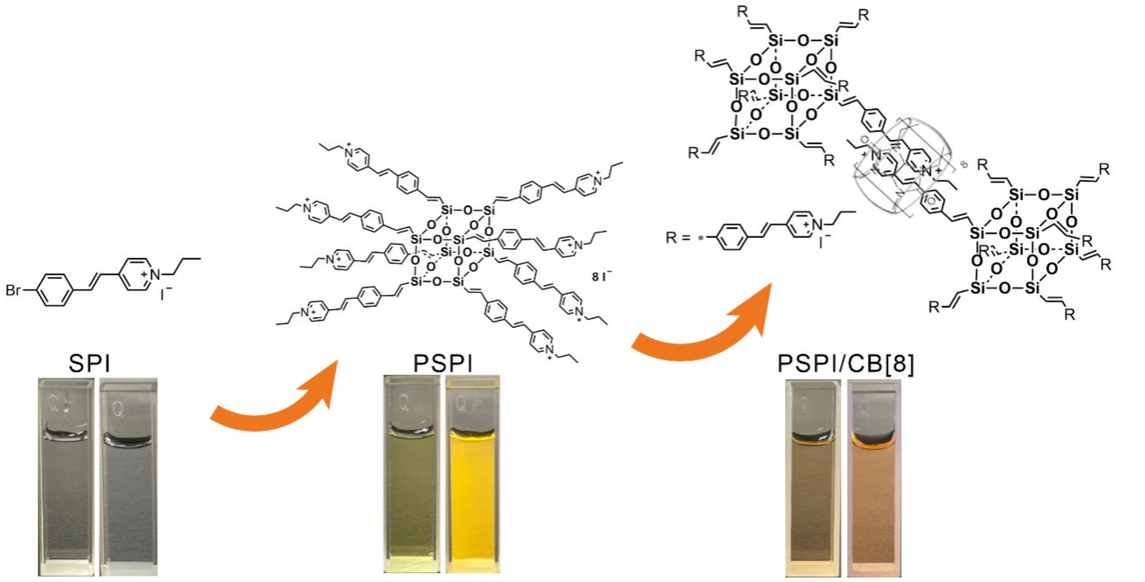

Figure S10. The chemical structures, pictures under naked eye (left part) and UV irradiation (365 nm, right part) of SPI, PSPI and PSPI/CB[8] assemblies (ACTI), respectively.

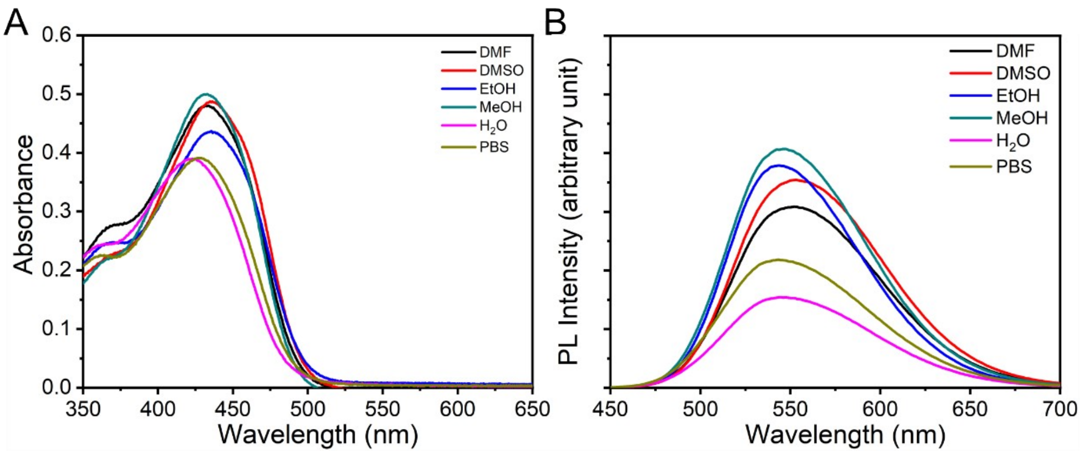

Figure S11. (A) The UV-vis absorbance and (B) PL intensity of PSPI in different solvents.

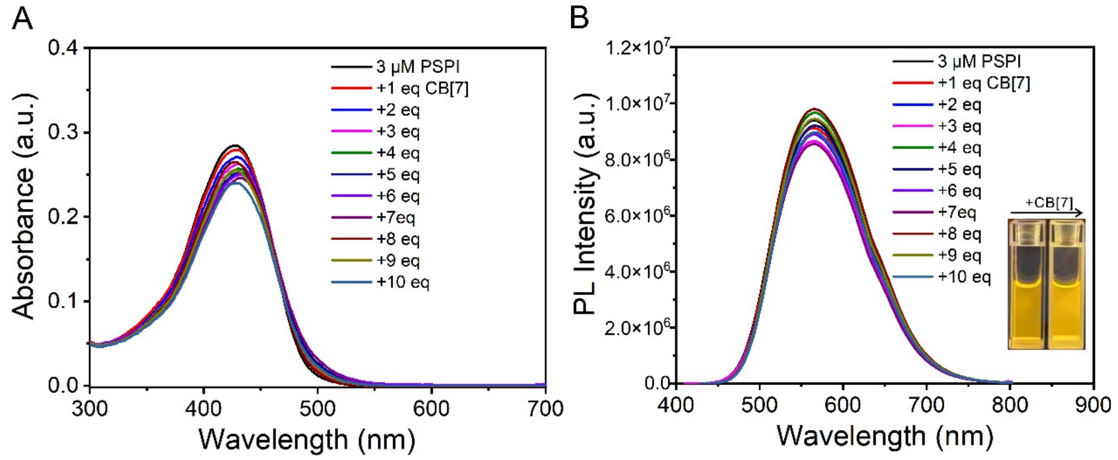

Figure S12. (A) The UV-vis absorbance and (B) fluorescence spectra of PSPI upon adding CB[7], the inset image is the photo of PSPI (left part) and PSPI/CB[7] (right part) under a UV 365 nm lamp.

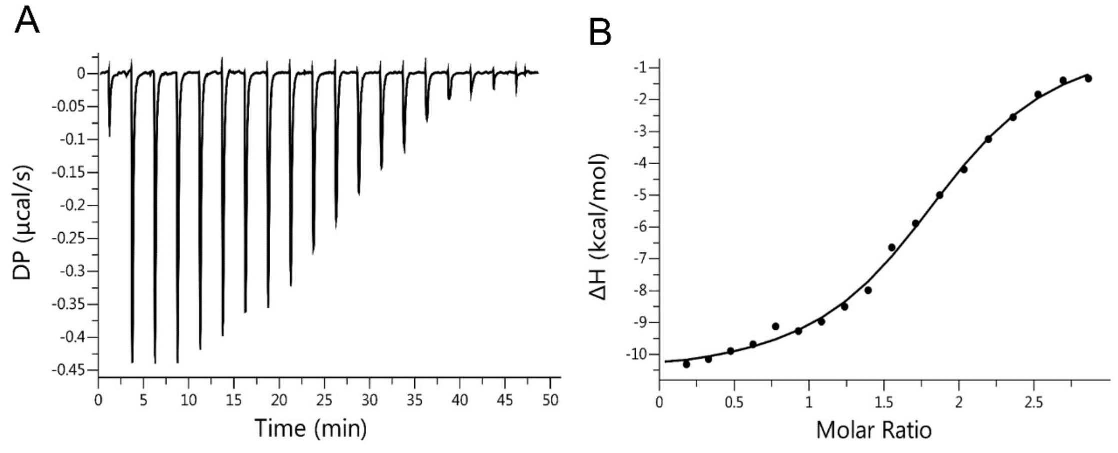

Figure S13. Isothermal titration calorimetry (ITC) of SPI with CB[8]. CB8[8][cell] = 20 μM, SPI[Syr] = 300 μM, N (sites) = 1.86±0.04, Ka = $5.4*10^5$ M^{-2}.

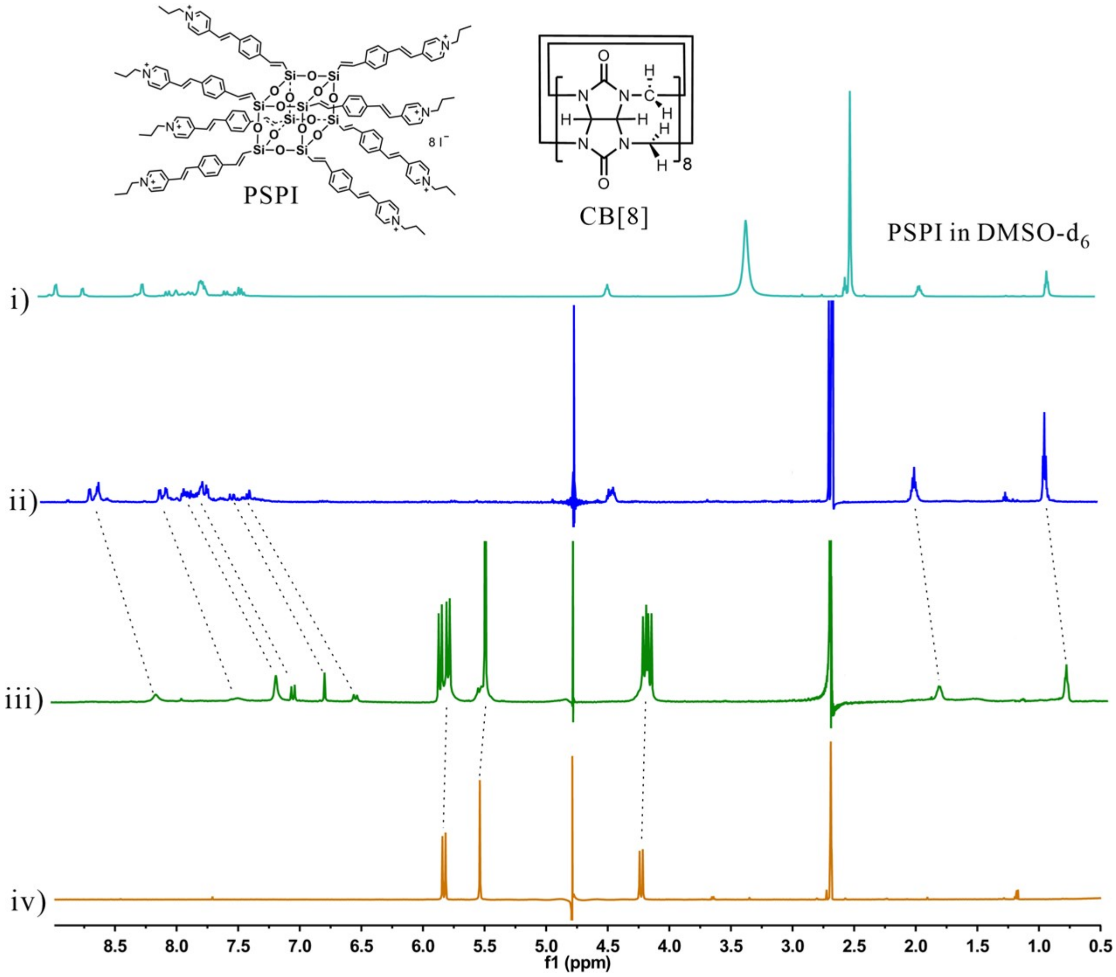

Figure S14. ^{1}H NMR spectra of (i) free PSPI in DMSO-d_6, (ii) free PSPI in D_2O/DMSO-d_6 (v/v, 9/1), (iii) PSPI with the addition of 5 equiv. CB[8] in D_2O and (iv) free CB[8] in D_2O /DMSO-d_6 (v/v, 9/1). About 10% DMSO-*d6* was added into (ii) and (iv) to meet the NMR test requirements and obtain high quality spectrum. Note: efficient water suppression approaches were implemented on these NMR titration procedures. Due to the unique nature of the spatial three-dimensional architecture of the octavinyl POSS skeleton, PSPI possess eight spatial orientated SPI arms. As such the aryl region of PSPI is crowded and the spectra exhibit oligomer-like properties, making its proton signals in the aromatic region broad and very close to each other in the ^{1}H NMR spectrum using D_2O as the solvent. The ^{1}H NMR titration between PSPI and CB[8] can only be approximated, and it is also difficult to assign each group of peaks in the PSPI/CB[8] assemblies.

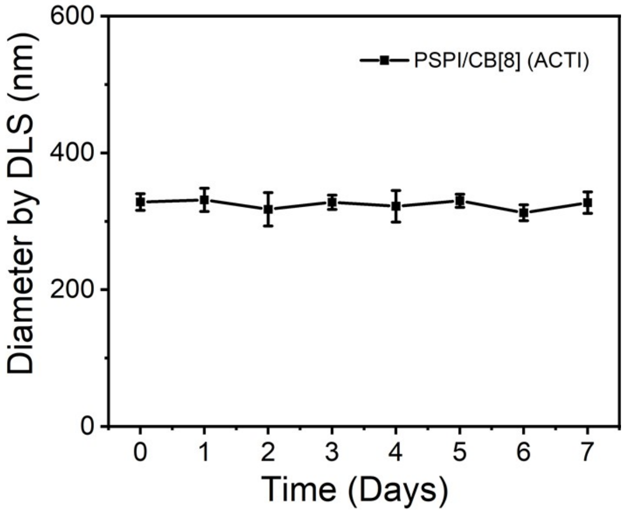

Figure S15. Stability of the PSPI/CB[8] assemblies in aqueous solution determined by DLS.

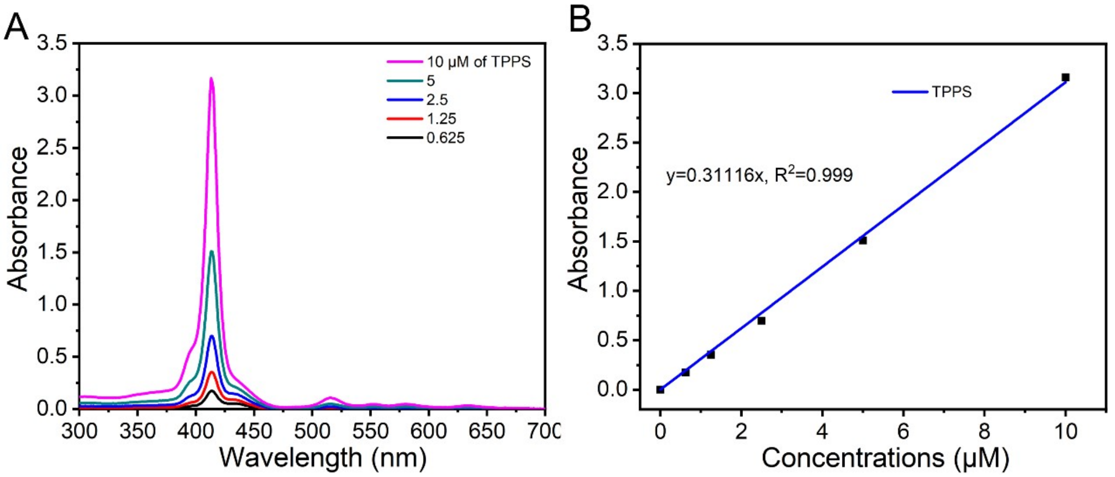

Figure S16. (A) The UV-vis absorbances of varied concentrations (0, 0.625, 1.25, 2.5, 5 and 10 μM) of TPPS in aqueous solutions, and (B) the standard curve based on the absorbance of TPPS at 413 nm.

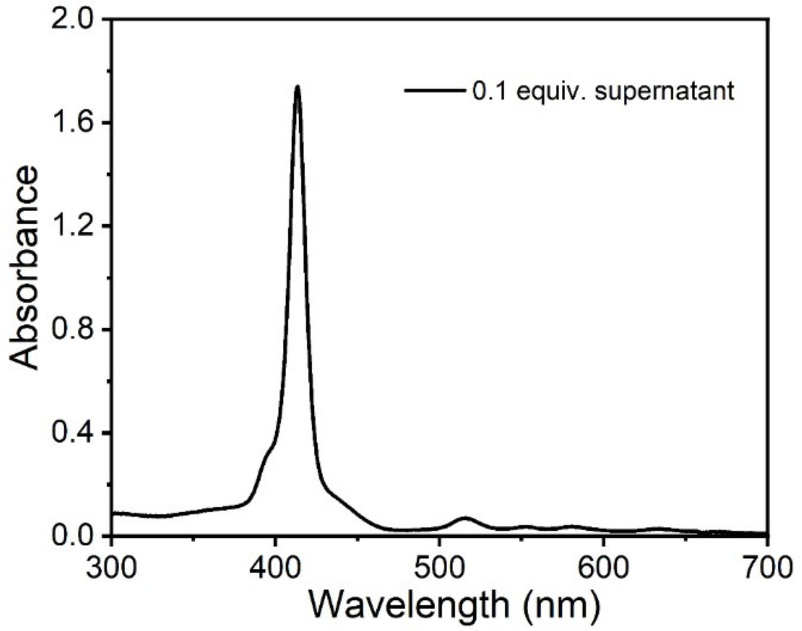

Figure S17. The UV-vis absorbance of the supernatant that diluted 10 folds. By measuring the absorption of supernatant diluted 10 fold, the unloaded TPPS could be calculated (concentration: 55.9 μM) according to the standard curve of TPPS (Figure S17). The loading content of TPPS onto ACTI can be determined by subtraction TPPS in supernatant from the total added. Finally, the loading efficiency of TPPS on TPPS@ACTI assemblies can be calculated to be 64.1%, meaning about 10 μmol TPPS@ACTI contains 6.41 μmol TPPS.

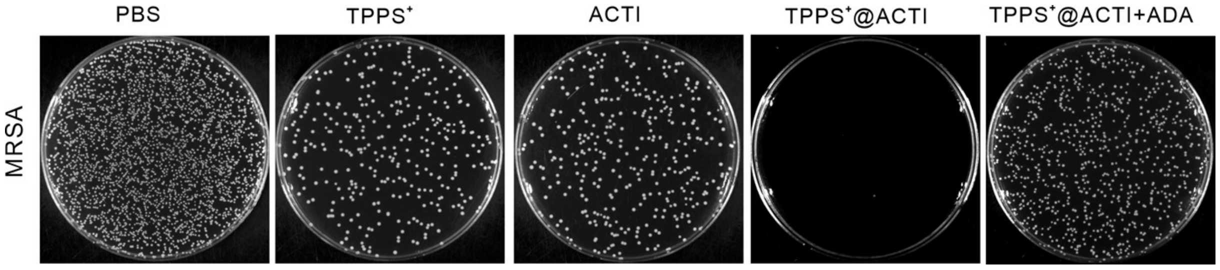

Figure S18. (D) Representative photographs of LB agar plates for MRSA with different treatments.

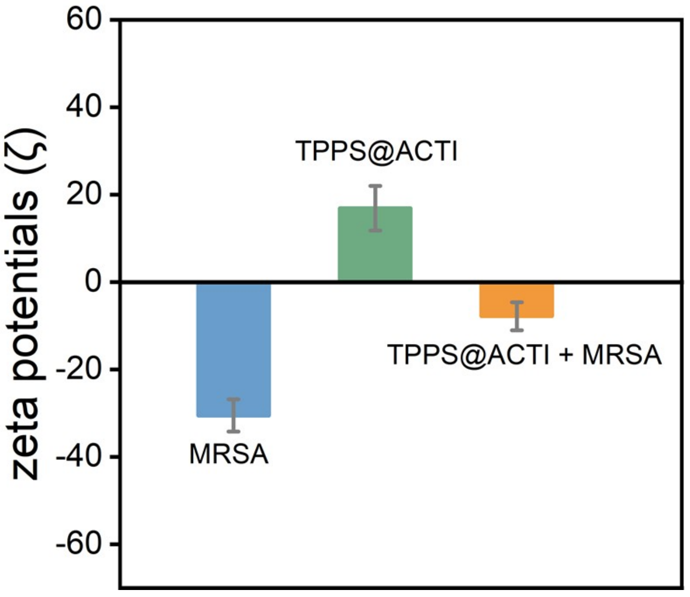

Figure S19. The zeta potentials (ζ) of MRSA, TPPS@ACTI and the mixture of MRSA treated with TPPS@ACTI.

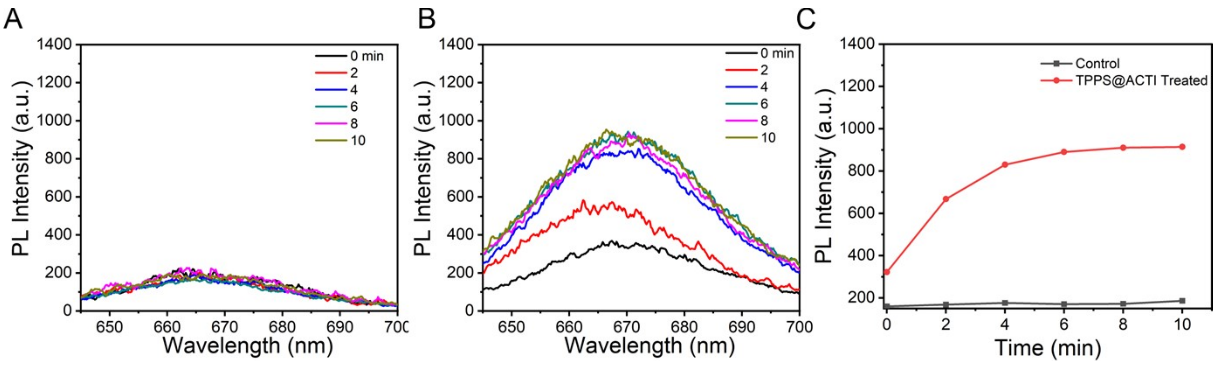

Figure S20. The PL intensity of DiSC3(5) treated by PBS (A, control group) and TPPS@ACTI (B). (C) Changes in fluorescence intensity of DISC3(5) with time, reflecting membrane potential disruption upon TPPS@ACTI treatment.

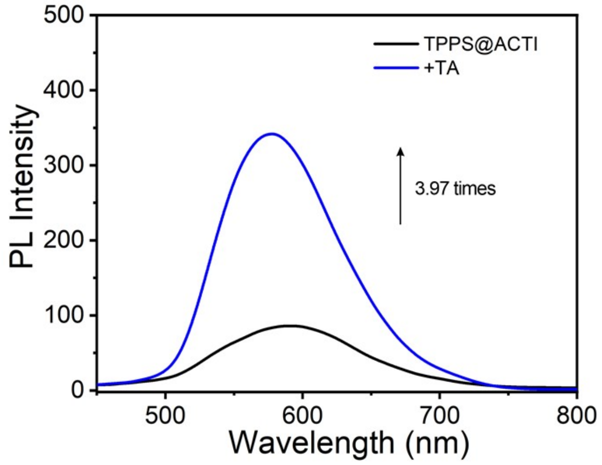

Figure S21. The fluorescence intensity of TPPS@ACTI and upon adding teichoic acid (TA).

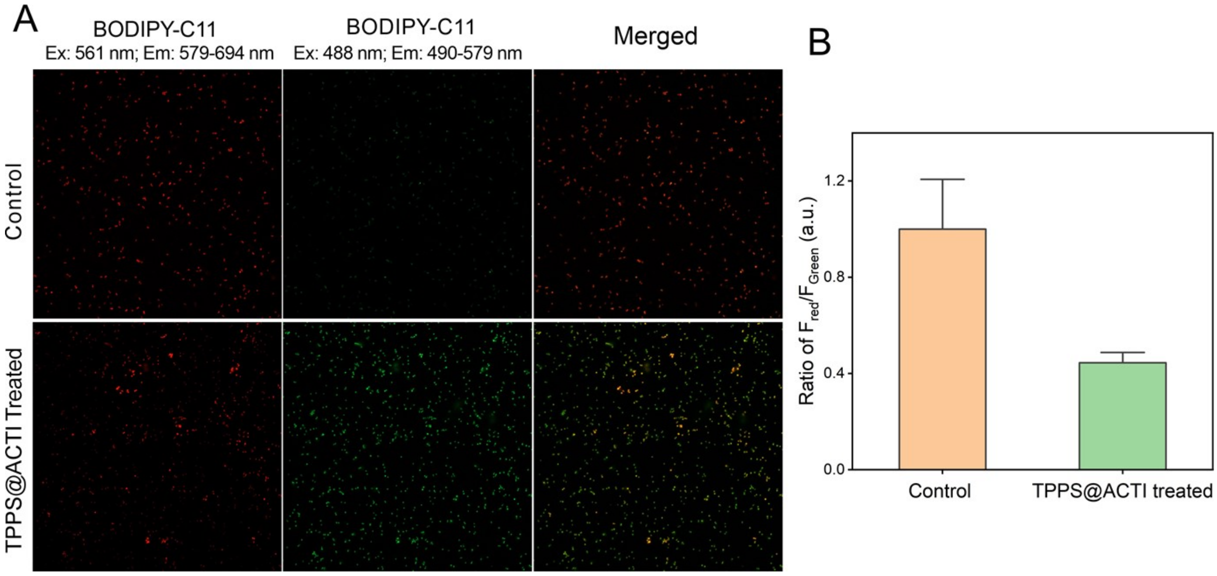

Figure S22. The CLSM images of MRSA treated with PBS (control group) and TPPS@ACTI, respectively, fluorescence of BODIPY-C11 in a reduced state (Red fluorescence) and oxidized state (green fluorescence). (B) The ratio of $F_{red/}F_{green}$ under different treatments.

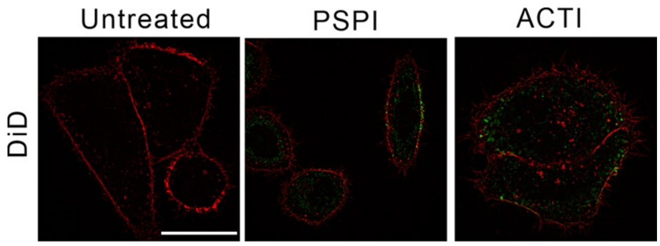

Figure S23. (A) Structure Illumination Microscopy images of HepG2 cells treated by PSPI and ACTI, PSPI and 2 μM ACTI at 37°C for 3h, respectively. PSPI, ex: 488 nm, em: 500-550 nm; ACTI, ex: 488 nm, em: 540-590 nm; DID, ex:639 nm, em: 647-694 nm. All the cells were stained by DiD for co-localization analysis. Scale bars: 20 μm.

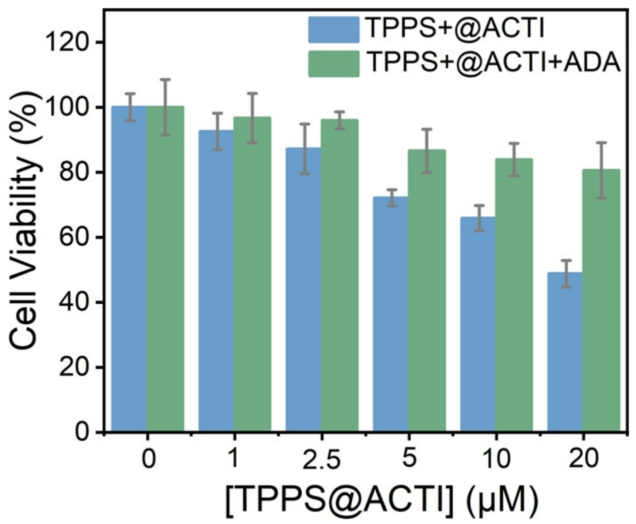

Figure S24. The cell viabilities of NIH/3T3 fibroblasts treated by different concentrations of TPPS@ACTI and upon adding a competing guest ADA.

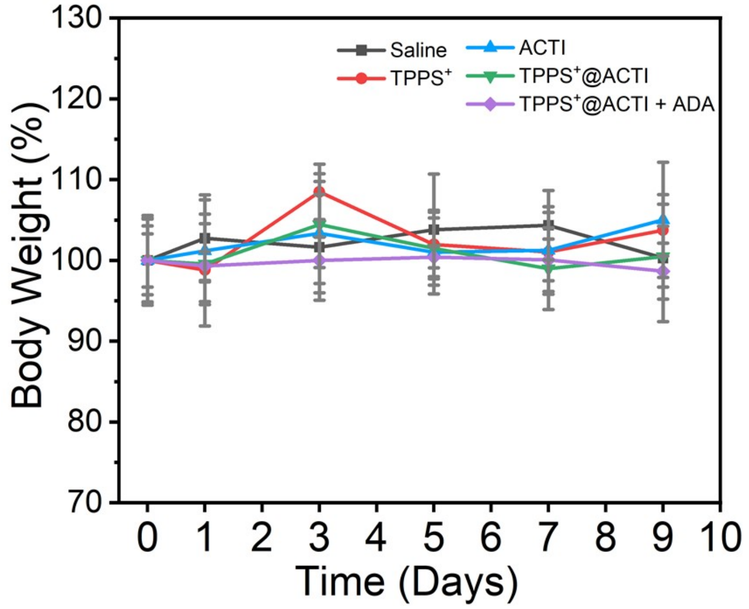

Figure S25. (A) Time-dependent mice weight curves after different treatments.

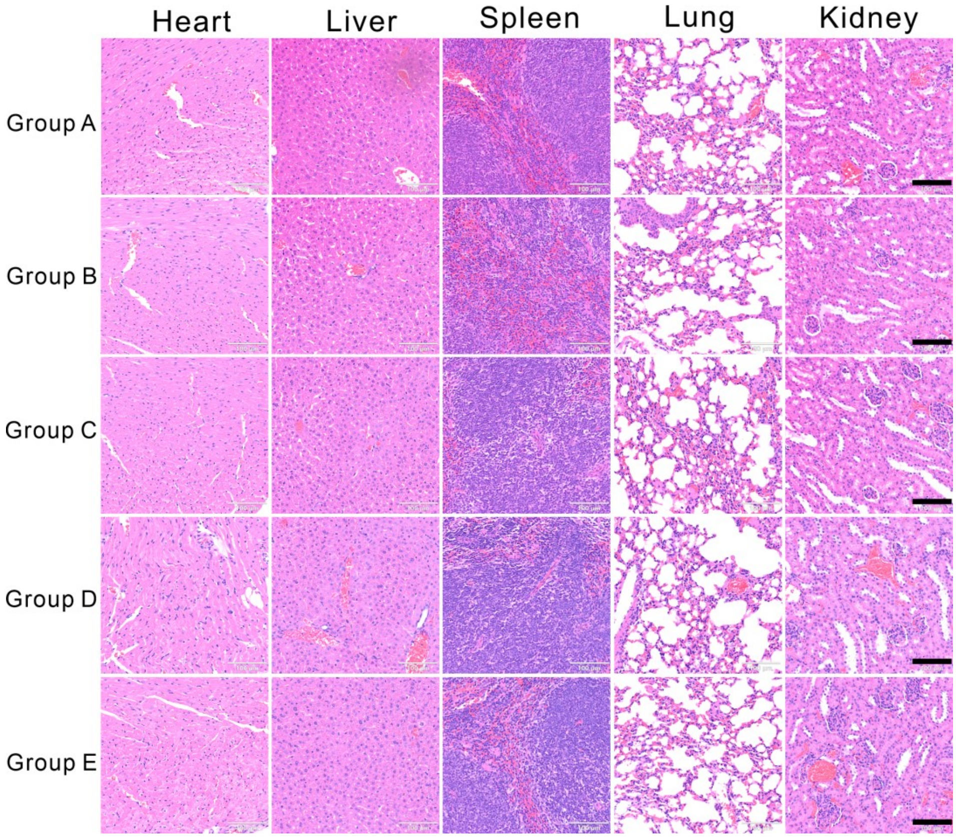

Figure S26. Photomicrographs of hematoxylin and eosin (H&E) stained tissues of mice main organs from different groups at Day 14. Scale bars, 100 μm.

16 References

1 Xing C, Xu Q, Tang H, Liu L, Wang S. Conjugated Polymer/Porphyrin Complexes for Efficient Energy Transfer and Improving Light-Activated Antibacterial Activity. *J. Am. Chem. Soc.* 2009, **131**, 13117-13124.

2 Chen J, Shan J, Xu Y, Su P, Tong L, Yuwen L, Weng L, Bao B, Wang L. Polyhedral Oligomeric Silsesquioxane (POSS)-Based Cationic Conjugated Oligoelectrolyte/Porphyrin for Efficient Energy Transfer and Multiamplified Antimicrobial Activity. *ACS Appl. Mater. Interfaces* 2018, **10**, 34455-34463.

3 Pu KY, Li K, Zhang X, Liu B. Conjugated oligoelectrolyte harnessed polyhedral oligomeric silsesquioxane as light-up hybrid nanodot for two-photon fluorescence imaging of cellular nucleus. *Adv. Mater.* 2010, **22**, 4186-4189.

4 Pu KY, Li K, Liu B. Cationic oligofluorene-substituted polyhedral oligomeric silsesquioxane as light-harvesting unimolecular nanoparticle for fluorescence amplification in cellular imaging. *Adv. Mater.* 2010, **22**, 643-646.

5 Horner S, Knauer S, Uth C, Jost M, Schmidts V, Frauendorf H, Thiele CM, Avrutina O, Kolmar H. Nanoscale Biodegradable Organic-Inorganic Hybrids for Efficient Cell Penetration and Drug Delivery. *Angew. Chem. Int. Ed.* 2016, **55**, 14842-14846.
